# A Proof-of-Concept for Safety Evaluation of Inhalation Exposure to Known Respiratory Irritants Using In Vitro and In Silico Methods

**DOI:** 10.3390/toxics13010035

**Published:** 2025-01-04

**Authors:** Nikaeta Sadekar, Holger Peter Behrsing, Tanja Hansen, Vivek Patel, Hazel Paulo, Alex Rae, Detlef Ritter, Katharina Schwarz, Anne Marie Api

**Affiliations:** 1 Research Institute for Fragrance Materials, Inc., Mahwah, NJ 07420, USA; amapi@rifm.org; 2 Institute for In Vitro Sciences, Inc., Gaithersburg, MD 20878, USA; hbehrsing@iivs.org (H.P.B.); vpatel@iivs.org (V.P.); 3 Fraunhofer Institute for Toxicology and Experimental Medicine ITEM, 30625 Hannover, Germany; tanja.hansen@item.fraunhofer.de (T.H.); detlef.ritter@item.fraunhofer.de (D.R.); katharina.schwarz@item.fraunhofer.de (K.S.); 4 Charles River Laboratories Edinburgh Ltd., Elphinstone Research Centre, Tranent EH33 2NE, UK; hazel.paulo@crl.com (H.P.);

**Keywords:** in vitro, in silico, exposure assessment, safety assessment, inhalation, threshold of toxicological concern

## Abstract

There is increased interest in developing non-animal test systems for inhalation exposure safety assessments. However, defined methodologies are absent for predicting local respiratory effects from inhalation exposure to irritants. The current study introduces a concept for applying in vitro and in silico methods for inhalation exposure safety assessment. Three in vitro systems, representing the upper (MucilAir™—nasal epithelial tissue) and lower (A549 cells and human precision-cut lung slices) human respiratory regions, were exposed to six respiratory irritants. These irritant exposures were conducted as liquid droplets, aerosol, or vapors, and samples were collected over 24 h. Cytotoxicity, cytokine release, epithelial resistance, oxidative stress, and mitochondrial membrane potential were measured. To determine the human relevance of in vitro exposures, airway surface depositions were predicted by simulating airborne concentrations equivalent to the Cramer class III inhalation threshold of toxicological concern limit of 0.47 mg/person/day using an in silico model. A > 100-fold margin of exposure was calculated comparing lowest concentrations showing in vitro effects to in silico simulated values. While further studies are needed, this manuscript presents a basic requirement for employing non-animal methods to inform inhalation exposure safety assessments by combining in vitro and in silico assays.

## 1. Introduction

In humans, acute exposures to irritant chemicals in the air can cause rhinorrhea, cough, pain, and bronchospasm, whereas chronic inhalation exposures may lead to chronic cough and rhinitis, non-allergic asthma, and reactive airway dysfunction syndrome (RADS) [1,2,3]. Such adverse effects of inhalation exposures in humans have a significant impact on economic productivity and health costs [4,5]. Thus, evaluating the effects from inhalation exposure to various compounds is critical in respiratory toxicity safety assessments. Traditionally, evaluation of inhalation exposure to cosmetic ingredients for local respiratory effects relied on toxicological studies using animals, even though there are limited in vivo inhalation exposure data, specifically on the irritant effects of inhaled chemicals in the respiratory tract [6,7]. This limited availability of inhalation exposure data may be attributed to the many challenges associated with conducting inhalation exposures in animal models potentially due to technical issues or failure to represent human pathological conditions [8,9,10]. Many of these issues may be mitigated, to an extent, by in vitro respiratory models, which expanded their role to be more precise and human-relevant despite the narrow range of their applicability.

In vitro models can be used to obtain a mechanistic understanding of tissue responses in airways following exposure to inhaled chemicals. However, owing to the dynamic nature of the respiratory system in vivo, there are some limitations related to physiology and exposure parameters (i.e., mode and duration of exposure) with in vitro respiratory models [11]. As observed from the in vivo inhalation studies, respiratory irritant responses are often complex and varied because of the involvement of sensory neuronal responses, which are absent from the existing in vitro models. Nonetheless, using isolated human primary tissues and immortal cell lines does provide a means to measure whether irritants induce local cellular damage in the respiratory tract. Variations in tissue composition also introduce uncertainties about their relevance in humans to local deposition and toxicological responses within the respiratory tract. This limits the use of any individual in vitro system for safety assessment. Due to the biological and functional diversity within tissues and cells of respiratory airways, in vitro models have been designed to address specific research questions. As such, it is important to consider their fit-for-purpose nature [11]. It is equally important to consider the nature of inhaled chemicals and their predicted depositions within the respiratory tract. Some chemicals, such as volatile organic compounds, are incompatible with aqueous in vitro cultures. Therefore, test chemical characteristics may influence the choice of in vitro models/systems and the choice of appropriate positive and negative controls.

Another challenge of using respiratory in vitro models is analyzing the data for their human relevance, which is complicated due to the dynamic nature of the respiratory system. Recent publications presented various refined approaches for deriving human-relevant points of departure (POD) from in vitro exposures to different irritant chemicals [12,13,14,15]. Some publications highlight the use of in silico prediction models, such as computational fluid–particle dynamics (CFPD), to calculate a human equivalent concentration using the in vitro benchmark dose lower confidence limit (BMDL) and the deposition of the chemical of interest in terms of the quantity and the specific region within the human upper respiratory tract [16,17]. In a retrospective analysis, authors have shown that the mean difference between data obtained from in vitro methods and threshold of toxicological concern (TTC) predictions developed for systemic effects was around 100-fold, and it is close to the established adjustment factor applied for translating animal data to humans to calculate the margin of safety in risk assessment [18]. However, whether similar results can be expected when applied to volatile or difficult-to-test compounds with specific exposure scenarios remains to be determined. Separate inhalation TTC values for local respiratory and systemic effects from a dataset of 92 chemicals with quality in vivo inhalation studies are reported by Carthew et al. [19]. A similar difference between inhalation TTC and in vitro-derived PODs would confirm the applicability of in vitro methods and the utility of the inhalation TTC, as an exposure-based waiving approach, for the safety assessment of these difficult-to-test compounds. Therefore, it is becoming increasingly clear that successful utilization of non-animal models requires a battery of in vitro and in silico assays to inform safety decisions.

The complex physico-chemical characteristics of some inhaled airborne irritants make it challenging to test them in relevant in vitro models utilizing traditional methods. Additionally, inhalation exposures in humans may account for the entire range of the compound’s physical and chemical properties. For example, exposures from semi-volatile compounds may consist of droplets and vapor phases simultaneously (aerosols). This further complicates the existing issue of dosimetry within the respiratory tract. A preliminary blind study was designed to gauge the sensitivity of three different in vitro respiratory models to known respiratory irritants. The study involved short-term single exposures to six known respiratory irritants with varying physico-chemical properties in three in vitro models representing different regions within the human respiratory tract, namely, MucilAir™, human precision-cut lung slices (hPCLS), and the A549 human alveolar epithelial cell line [13,20,21]. These in vitro models were selected since they represent the upper and lower respiratory regions. Subsequently, the analysis was expanded to include a preliminary exercise comparing in vitro data with inhalation TTC limits to establish the human relevance of the observations obtained from these non-animal methods. To enable this comparison, a human exposure concentration equivalent to the Cramer class III (CCIII) inhalation TTC limit of 0.47 mg/person/day [19] was simulated using the modified vapor uptake multiple-path particle dosimetry (MPPD) model developed for refining fragrance exposures [22,23]. Due to the strategic importance of inhalation TTC as an animal-alternative method, the most conservative CCIII inhalation TTC value was chosen for this purpose [24]. When these predicted values are compared to the PODs identified in the in vitro experiments, the results present a combined in vitro/in silico approach as a promising avenue for conducting NAM (non-animal method)-based inhalation exposure risk assessments. Although variable responses were observed in the in vitro models, this proof-of-concept study demonstrates that their utility in informing safety conclusions depends on the model’s fitness for purpose and the human relevance of the biomarkers.

## 2. Materials and Methods

### 2.1. Test Chemicals

Acetic acid (CAS No. 64-19-7), ammonium hydroxide (CAS 1336-21-6), benzyl isocyanate (CAS 3173-56-6) or trimellitic anhydride (TMA, CAS 552-30-7), capsaicin (CAS 404-86-4), formaldehyde (CAS 50-00-0), toluene (CAS 108-88-3), lactose (CAS 63-42-3), and salicylic acid (CAS 69-72-7) were either purchased from Millipore Sigma (St. Louis, MO, USA) or Merck KGaA (Darmstadt, Germany). Triton™ X-100 (CAS 9002-93-1) was purchased from Thermo Fisher Scientific (Waltham, MA, USA). Sodium dodecyl sulfate (SDS, CAS 151-21-3) was purchased from Carl Roth (Karlsruhe, Germany). Due to issues with chemical stability and compatibility with tissue culture materials, benzyl isocyanate was replaced with trimellitic anhydride for the A549 cell model. The test chemicals were supplied with appropriate certificates of analysis indicating their purity. The identities of the test chemicals were masked during study execution, data collection, and analysis and were unmasked for finalizing the study report.

### 2.2. In Vitro Respiratory Models

MucilAir™ nasal epithelium of a single donor and MucilAir™ culture medium was obtained from Epithelix Sàrl, 18 Chemin des Aulx, CH 1228 Plan-Les-Ouates, Geneva, Switzerland. MucilAir™ is an in vitro tissue model of the human upper airway epithelium cultured at the air–liquid interface (ALI). It is a powerful and predictive model for in vitro research and tests. MucilAir™ tissue is mainly composed of the following cell types: basal cells (progenitor cells), goblet cells (mucus-producing cells), and ciliated cells (with active cilia) (https://www.epithelix.com/products/mucilair, accessed on 7 September 2024).

Human precision-cut lung slice (hPCLS) [25] is a non-transplantable non-diseased human donor lung tissue-based ex vivo model obtained from the National Disease Research Interchange (NDRI; Philadelphia, PA, USA). The lung filling buffer used to inflate the lung contained 0.8% Agarose I (bioWORLD, Dublin, OH), HBSS (Gibco, Thermo Fisher Scientific, Waltham, MA, USA), DMEM:F-12 (1:1) medium (Lonza, Walkersville, MD, USA), 1% antibiotic–antimycotic (Gibco, Thermo Fisher Scientific, Waltham, MA, USA), 1% GlutaMAX™ (Gibco, Thermo Fisher Scientific, Waltham, MA, USA), and 10 µg/mL 2-phospho-L-ascorbic acid trisodium salt (Millipore Sigma, St. Louis, MO, USA). The slicing buffer used for slicing lung cores consisted of Belzer UW^®^ cold storage solution (Bridge to Life, Northbrook, IL, USA), 0.9 mg/mL reduced L-glutathione (Millipore Sigma, St. Louis, MO, USA), 0.01 mg/mL 2-phospho-L-ascorbic acid trisodium salt, and 1% antibiotic–antimycotic solution (Millipore Sigma, St. Louis, MO, USA). The hPCLS was cultured in DMEM:F-12 (1:1) medium containing 1% GlutaMAX™, 1% Insulin-Transferrin-Selenium-G (Gibco, Thermo Fisher Scientific, Waltham, MA, USA), and 1% antibiotic–antimycotic solution. The culture medium also contained 2 µM hydrocortisone (Millipore Sigma, St. Louis, MO, USA) during the initial acclimation period of 1–3 days to suppress the possible inflammatory responses due to the slicing procedures.

A549 air–liquid interface (ALI) cultures: The human A549 cell line (ATCC^®^CCL-185™) was purchased from the American Type Culture Collection (ATCC; LGC Promochem). The A549 cell model, an immortalized cell line of the human lung epithelium, was used because it is well characterized and the morphology and basic cellular functions, such as surfactant synthesis, oxidative metabolism, and transport properties, are consistent with those of alveolar epithelial type II cells [26]. Additionally, historical data available at Fraunhofer ITEM show a good correlation between acute toxicity data obtained in A549 cells at the ALI and in vivo inhalation data obtained in rats.

Methods employed in the study using six known respiratory irritants blind tested in three in vitro models are summarized in Table 1. Detailed information on in vitro culture conditions, experimental procedures, and statistical analyses is provided in Appendix A. The study used three in vitro respiratory models representing upper and lower respiratory tissues. The exposures were conducted at an air–liquid interface (ALI) by masking the identities of six known respiratory irritants with complex physico-chemical properties. Salicylic acid and lactose represent the ingredients used in the consumer products and they were, respectively, selected as positive and negative controls in this study for evaluating local irritant effects in the respiratory tissues [27,28,29]. Since salicylic acid is a relatively mild irritant, compared to the more potent SDS or Triton™ X-100 [13], the latter two chemicals were added as additional positive controls. The mode of exposure included small-volume direct application of test chemical solution (MucilAir™ model), aerosol exposures using DMSO (hPCLS model), and vapor or aerosol exposure of the pure substances from the airborne state (A549 model). The in vitro exposures for each test chemical were carried out with a range of concentrations and exposure durations. The resultant exposure-related effects were analyzed and compared to time-matched controls.

### 2.3. In Silico Simulations Using Multiple-Path Particle Dosimetry (MPPD) Model

The MPPD model that determines the depositions in the lower respiratory tract was used to simulate airborne concentrations equivalent to CCIII inhalation TTC value of 0.47 mg/day for local respiratory effects [19] for the seven test chemicals (including trimellitic anhydride). These simulations provided the rate of mass deposited per unit surface area. The physico-chemical properties of the materials used for simulating these exposures are summarized in Table 2.

## 3. Results

This proof-of-concept study provides a means for practical application of the in vitro and in silico methods towards inhalation exposure safety assessment. Specific details on the exposure-related observations from all three models are outlined below. The supplementary document provides background data used to summarize the results presented here.

### 3.1. MucilAir™

Table 3A,B summarizes the observations from the MucilAir™ model. Barrier integrity and IL-6 release were observed as critical markers of irritant exposure-related cytotoxic effects in the upper respiratory tissue model MucilAir™.

The positive controls salicylic acid and SDS (Table 3A) had no effect on LDH release. In contrast, a negative impact on barrier integrity (TEER) was observed at all time points. A > 2-fold increase in IL-6 release was also observed for the two positive controls at 24 h.

Viability measured by LHD release (Table 3A) was not affected by the irritant exposures. Formaldehyde exposures showed an exposure concentration and duration-related reduction in resazurin metabolism (Table 3B) indicating increased cell damage in MucilAir™.

TEER (Table 3A,B) was measured to capture the barrier integrity of the MucilAir™ tissues. All irritant exposures affected tissue barrier integrity, with the most severe effect observed from acetic acid and benzyl isocyanate exposures at all concentrations and time points.

IL-6 and IL-8 cytokine releases (Table 3A,B) were measured using ELISA. Greater than 2-fold increases in IL-6 release were observed at 24 h (182 and 273 µg/cm^2^ acetic acid, 182 µg/cm^2^ benzyl isocyanate, and 182 µg/cm^2^ toluene exposures), 6 h (23 µg/cm^2^ capsaicin and 136 µg/cm^2^ toluene), and 1 h (46 µg/cm^2^ capsaicin). Greater than 2-fold increases in IL-8 release were not observed from irritant exposures.

### 3.2. Human Precision-Cut Lung Slices (hPCLS)

Table 4 summarizes the observations from the hPCLS model. Overall, the effects on viability and cytokine release were important criteria for evaluating irritant exposures in the hPCLS model.

Salicylic acid and Triton™ X-100 significantly affected the hPCLS tissue viability at all time points. However, IL-8 release was observed at a > 2-fold relative increase at 1 and 6 h exposures.

All test chemical exposures affected viability, with ammonium hydroxide and formaldehyde showing significant effects at all concentrations and time points.

IL-6 and IL-8 releases were measured using ELISA. Greater than 2-fold increases in both cytokines were observed at 24 h (11 and 32 µg/cm^2^ capsaicin and 263 and 1842 µg/cm^2^ toluene), 6 h (18 µg/cm^2^ benzyl isocyanate, 32 and 95 µg/cm^2^ capsaicin, and 1842 µg/cm^2^ toluene), and 1 h exposures (32 µg/cm^2^ capsaicin). A 24 h exposure to benzyl isocyanate at 18 µg/cm^2^ showed a >2-fold increase in IL-6 alone.

### 3.3. A459 Cells

Table 5 summarizes the observations from the A549 cell model. All irritant chemical exposures affected cell viability, mitochondrial membrane potential (MMP), and unspecified cellular stress at 24 h except toluene vapor exposures, which resulted in the highest LC_50_ and LOAEL values observed for the measured endpoints. Vapor form exposures for the semi-volatile irritant chemicals were less potent compared to their respective aerosol forms. Unspecified cell stress was the most sensitive parameter in A549 cells, with small but persistent effects beginning from the lowest concentrations for irritant exposures.

The A549 cells were not affected by salicylic acid exposures (Supplementary Data, Figures S1b and S2b).

SDS exposures resulted in a concentration presenting 50% cell mortality (LC_50_) of 6.8 µg/cm^2^ with a low observed adverse effect level (LOAEL) for viability of 0.65 µg/cm^2^ at 24 h. The 24-hour LOAEL for mitochondrial membrane potential (MMP) was 4.94 µg/cm^2^, while the 24-hour LOAEL for unspecific cell stress was 0.22 µg/cm^2^ from SDS exposures.

Ammonium hydroxide aerosol exposures, followed by formaldehyde aerosol exposures, resulted in the lowest LC_50_ for viability and LOAEL values for MMP. Capsaicin, followed by trimellitic anhydride, showed the lowest LOAEL value for unspecified cellular stress effects.

To summarize, all in vitro respiratory models were found to be suitable for recognizing irritant exposures, but depending on the model, different biomarkers were observed as critical for local respiratory irritant effects evaluation.

### 3.4. In Silico Simulation Using MPPD

Due to the dynamic nature of the respiratory system, the concentrations at the site of exposure along the respiratory tract are vastly different from the inhaled airborne concentrations. As such, correlating the in vitro exposures to human inhalation exposure scenarios is impractical. Instead of comparing the in vitro test solution concentrations (mg/L) with human environmental exposure concentrations (ppm or mg/m^3^), a modified vapor uptake MPPD model was used to translate the environmental concentrations into a form comparable with the in vitro exposure concentrations (µg/cm^2^). The MPPD simulations were carried out using test chemical-specific physical and chemical properties (Table 2) to simulate 0.47 mg/person/day-equivalent (Cramer class III inhalation TTC value) airborne concentrations and in vitro vapor exposure concentrations (ppm) to convert to the amount deposited per unit surface area (µg/cm^2^). Although the computational model provided depositions for different generations of the lower respiratory tract, the highest deposition observed for a particular generation was used to represent these exposures.

For the hPCLS model, the PODs for each test chemical were identified based on the combination of the lowest concentration and shortest exposure duration showing a statistically significant biological effect (reduction in viability and/or increase in IL-6/IL-8 release; as presented in Table 4). POD for ammonium hydroxide and formaldehyde exposures were not identified due to near complete loss of viable tissue.

LOAELs from the A549 cell-based in vitro data were identified as the concentration values coinciding with the 95% confidence interval for the measured endpoints: viability, MMP, and unspecified cellular stress (as presented in Table 5). However, it is important to establish the human relevance of sensitive measures, such as unspecified cellular stress, from in vitro models. Therefore, the PODs considered for the A549 cell model were identified based on the lowest concentration showing a significant impact on relevant endpoints (viability and/or MMP).

The margin of exposure (MOE) was calculated for each test chemical considering the physical form of exposure and the most conservative POD identified between the hPCLS and A549 cell in vitro models. The following formula was used to calculate the MOE as the ratio of the deposited mass per unit surface area identified from in vitro exposures and MPPD-simulated human-equivalent exposures:

(1) MOE=in vitro POD (ugcm2)MPPD simulated deposition (ugcm2).

Together with the in vitro PODs (summarized in Table 6), the CCIII inhalation TTC-equivalent concentration was considered for calculating the margin of exposure (Table 7).

## 4. Discussion

The study is a proof-of-concept comparing in vitro exposures to the human-relevant MPPD-predicted site-specific depositions within the lower respiratory regions. The considerations made in this analysis align with the elements of the framework for building scientific confidence in non-animal methods as proposed by van der Zalm et al. [30]. The framework consists of five elements for determining the applicability of in vitro and in silico methods; namely fitness for purpose, human biological relevance, technical characterization, data integrity and transparency, and independent review. The discussion addresses the fitness for purpose and human biological relevance of the non-animal methods and highlights the need for technical characterization and data integrity and transparency of these methods in informing inhalation exposure safety assessments.

Since all the test chemicals are known respiratory irritants, positive and negative controls in the studies were used only to ensure that the exposures were effective. The test chemical identities were masked for carrying out experimental procedures in all three in vitro models to prevent bias. Some technical or procedural challenges were encountered due to the blinded nature of the study, including the suitability of the test chemicals for aerosol exposures due to their complex physico-chemical properties. For example, it is acknowledged that deposited material from aerosol exposures varies significantly for particles or droplets [31] and precise quantification for semi-volatile or difficult-to-test chemicals is not possible. The experimental outcomes presented here are meant to highlight the challenges faced in the standardization and validation of in vitro methods due to the nature of the test chemicals and the variability observed within in vitro models. The discussion outlines how these methods can be applied, once these challenges are resolved, to conduct inhalation exposure safety assessments supported by appropriate justifications and uncertainty considerations to establish their fitness for purpose.

In this study, only two in vitro models (hPCLS and A549 cells) have data with robust statistical evaluations. The data from the MucilAir™ model were obtained from only two experimental replicates, and therefore, not analyzed for statistical significance (detailed information on technical methods and statistical evaluations are provided under Appendix A). As such, data from MucilAir™ were not included in calculating the MOE. Since MucilAir™ tissues are an established functional model closely representing the human upper airway or nasal respiratory epithelium, the observations described herein provided valuable insights into the potential irritant exposure effects on the nasal epithelium. Additionally, damage to MucilAir™ by the model irritant, SDS, has been previously shown to result in measurable changes in TEER, LDH release, and resazurin metabolism [13].

No significant changes or similarities between trends were observed with respect to IL-1β, MCP-1, TNF-α, GM-CSF release, and oxidative stress, so data from these endpoints are not presented here. Overall, barrier integrity and IL-6 releases were identified as critical markers of upper respiratory irritation in MucilAir™, while viability combined with IL-6 and IL-8 releases was important for evaluating irritant exposure effects in the lower respiratory region using the hPCLS model. Contrastingly, irritant chemical exposures in A549 cells demonstrated a reduction in viability with no increases in IL-6 and IL-8 cytokine release. A similar observation was published by Standiford et al., suggesting that inter-cellular cross talk, particularly between activated alveolar macrophages and type II pneumocyte-like epithelial cells, facilitates IL-8 release from A549 cells [32]. In the present study, additional endpoints, such as effects on MMP as a marker for oxidative stress and unspecified cellular stress, were included for evaluating irritant effects in A549 cells.

Measuring cytokine releases, such as IL-6 and IL-8, together with other cytotoxicity endpoints depending on the in vitro model characteristics, are standard approaches for local respiratory irritation assessment from irritant chemical exposures [13,15], and therefore, these were included in the current study as a measure of irritant exposure-related effects. These studies also evaluated either a single in vitro respiratory model or compared different models representing the same respiratory region [13,15]. To our knowledge, the current study is the only assessment where in vitro models representing upper and lower respiratory regions were evaluated simultaneously. It is apparent from the data presented here that depending on which region within the respiratory tract is represented by the in vitro model, tissue responses to irritant exposures are different. This implies that different sets of endpoints define local respiratory irritation for different regions within the respiratory tract.

As a result, a single model may not be predictive for extrapolating local respiratory irritant effects to the whole respiratory system. Moreover, it is important to establish the human relevance of the in vitro observations. As test models and methods are developed further, they may become more refined and their sensitivity increased to capture the slightest perturbations in the test system. For example, in the current study, a non-traditional endpoint (unspecified cellular stress) was used in the A549 cell model, which was observed to be the most sensitive of the endpoints considered for evaluating irritant exposures. However, with limited information available from human exposures, it is difficult to determine the biological relevance of such data.

Studies with ammonia exposures up to 100 ppm in humans showed that the effects were adaptable over time and did not affect the overall health of the volunteer subjects [33,34]. Due to the vast difference in the site-specific exposure concentrations upon inhalation and the airborne concentrations in the breathing environment, a direct comparison with the A549 model-derived LOAEL values is not practical. It is also observed that humans appear particularly susceptible to extra-thoracic or upper respiratory effects compared to the observations in animals [33,34,35,36]. For example, the odor irritation threshold in humans for formaldehyde exposures is reported at 1.22 ppm [35]. A surface-level examination of formaldehyde data from the A549 cell model and the human exposures makes the A549 cell in vitro inapplicable for informing on safe human exposures. As such, a direct comparison of the in vitro concentrations and human exposures, especially for those with complex physico-chemical properties, requires multiple analytical approaches consisting of both in vitro and in silico models.

The MPPD is a modified vapor-uptake in silico model, used to simulate inhalation exposures to a chemical in its vapor and or aerosol form and determine the airway depositions of the inhaled chemical in the lower respiratory regions [22]. The MPPD simulations provided exposure values in terms of the rate at which the amount of material is deposited per unit surface area in a specific region of the lower respiratory tract in humans. In this study, the highest deposition observed within a specific generation of the lower respiratory tract was used to calculate the MOE. For an effective comparison, certain assumptions were made that may render these evaluations more conservative. It was assumed that 100% of the exposure concentrations simulated in the MPPD model were inhalable, and the clearance from the airway tissues by mucociliary action, metabolism, and absorption was not included in these in silico determinations. These assumptions bring the simulated exposure closer to the in vitro scenario where the test compound is introduced directly on the apical side of the culture which lacks the many clearance mechanisms presented in the human airways. The human relevance of these in silico simulations is achieved by accounting for the dynamics of human respiration. For example, the model incorporates standard values representing healthy adult humans, such as 3300 mL functional residual capacity (FRC) and 50 mL upper respiratory tract (URT) volume, assuming 12 breaths per minute and 750 mL tidal volume (TV). Due to this dynamic feature, the exposures often did not reach the pulmonary or alveolar region when simulating human-relevant inhalation exposure concentrations. Table 7 summarizes the outcomes from MPPD simulations and MOE calculated by comparing the inhalation TTC-equivalent exposures and in vitro PODs.

It is worth noting that obtaining relevant outputs from the MPPD simulations requires using either predicted or measured values for specific physical and chemical parameters. In other words, if a measured value is not available for even a single parameter for a particular chemical, predicted values should be used for all the parameters for that chemical. Exposures for all test chemicals from this study were simulated using predicted physico-chemical properties [37,38], except for formaldehyde vapor simulation, which used measured values (Table 2). Since the predicted values tend to differ from experimentally measured ones, some variation is expected in the results [39]. Consideration of uncertainty factors is required to compensate for the associated variability. For example, concentrations derived from aerosol exposures in vitro are estimations of the test chemical deposited on the apical surface of the culture. The MOEs calculated in Table 7 do not account for the anticipated uncertainties and variability. However, without adjusting for variability in tissue responses and uncertainties involved in data translation, the in vitro-derived PODs were generally observed to be 100-fold greater than the CC III inhalation TTC-derived PODs. The consideration of a greater than 100-fold difference is based on a 10-fold component for the variability in responses between human tissues in vitro and a 10-fold component for in vitro to in vivo data translation, similar to the in vivo adjustment factors for inter- and intra-species variability. Paul Friedman et al. [18] reported a 100-fold difference between NAM-derived and TTC-derived PODs for systemic effects when compiling data from 448 substances for undefined chemical space and different portals of entry. Data generated from testing six known irritants in the three respiratory in vitro models integrate very well into the concept presented by Paul Friedman et al. [18], even though they were derived for a specific testing scenario, i.e., inhalation exposures to known irritants with complex physico-chemical properties. As observed, the results confirm the integrity of the NAM and the TTC concepts for local respiratory toxicity evaluation and potentially deliver valuable information for safety assessment strategies. Thus, the results of this exercise show that the toxicity ranking in terms of irritant potency for aerosol exposures was ammonium hydroxide > formaldehyde > acetic acid > trimellitic anhydride > capsaicin > benzyl isocyanate > toluene. Similarly, irritant potency observed in vapor exposure was formaldehyde > acetic acid > ammonium hydroxide > toluene.

As stated previously, due to the dynamic nature of the respiratory system the concentrations along the respiratory tract vary significantly from the inhaled airborne concentrations. Using an in silico model to determine surface depositions in the respiratory tract is an important step for facilitating direct comparison between human exposures and in vitro exposures. Ramanaraynan et al. published the very first case study for NAM-based inhalation exposure safety assessment [17]. The authors presented a framework involving the use of CFD in silico model to determine the airway depositions which were used for in vitro model exposures using MucilAir™. By using BMD modeling, the authors identified a POD from in vitro exposures which was converted to a human equivalent concentration (HEC) representing the potential irritation inducing airborne concentration. The current study proposes a refined framework for inhalation exposure safety evaluation by incorporating at least two in vitro models representing upper and lower respiratory regions and a suitable in silico model to simulate human-relevant inhalation exposure concentrations. Determining airway depositions from human-relevant exposures allows for a direct comparison with in vitro PODs, thus avoiding compounded uncertainty due to multiple mathematical manipulations of the data.

Developing this proof-of-concept study and its application came with many challenges. Highlighted below are the insights gained from these studies for future consideration.

Consideration of the physical and chemical parameters of a test chemical is essential when defining study design and mode of exposure. It is recommended that in vitro exposures to semi-volatile chemicals be limited to direct liquid application and short-term duration. If possible, include separate testing of the pure vapor form to enable distinct dosimetric interpretation of effects from the condensed form.

Using an in silico tool, such as the modified vapor uptake MPPD model [22], may be required at the study design stage and also for calculating the MOE. However, the in vitro aerosol/vapor exposures need to be well-characterized. A Quartz Crystal Microbalance (QCM) is utilized for quantifying deposited mass in the ALI cultures from aerosol exposure systems. Since it is a challenge for characterizing such exposures for semi-volatile or difficult-to-test substances, it is recommended to employ direct small-volume liquid applications for reducing dosimetric uncertainties.

The diversity of the respiratory tract shows region-specific differences in inflammatory responses. As such, inhalation exposure safety assessments for the portal of entry effects may necessitate testing at least two in vitro models, representing upper and lower respiratory regions.

A combination of endpoints and biomarkers for any single model may be critical for drawing safety conclusions depending on the nature of the test chemical. For example, a combination of the effect on viability, cytokine release, barrier integrity (upper respiratory), and/or MMP measurements may be required for deriving safety conclusions for acute inhalation exposure to irritants.

Due to the variability observed in the tissue responses, it was not possible to identify trends across the three models even when using the same test chemicals. For example, increased cytokine release was observed in MucilAir™ and hPCLS but not in A549 cells.

Increased cytokine release was not always observed at sub-cytotoxic concentrations. This indicates that there is a need to integrate scenarios involving human exposure to irritant chemicals to help solidify the biological relevance of cytokine measurements in in vitro models. This, in turn, reinforces the need for additional work to characterize and standardize the in vitro and in silico models, establish the clinical relevance of the endpoints, and the importance of detailed documentation of the test procedures.

## 5. Conclusions

The study demonstrated that in vitro testing of a small set of reference substances resulted in PODs that were higher than the inhalation TTC-derived values. In summary, this study highlighted that (a) the mode of exposure is dependent on the physical and chemical properties of the test chemical, (b) suitable biomarkers of irritation are dependent on the in vitro model used for evaluating respiratory irritation, and (c) loss of viability (lower respiratory region) and barrier integrity (upper respiratory region) combined with other markers such as damage-associated molecular patterns are important for local respiratory irritation assessment. The study also highlighted the consideration of uncertainty factors and the need for additional research to standardize this testing approach, as it shows that local respiratory evaluations require multiple in vitro models combined with suitable in silico models. The current work demonstrated a high value and promising perspective for applying NAMs in consumer products’ exposure assessments, which may be further elaborated for determining protective exposure concentrations by incorporating a larger number of model substances for ingredients in consumer products.

## Figures and Tables

**Table 1:** Brief description of the evaluation of three in vitro respiratory models using six known respiratory irritants.

Methods	MucilAi™	hPCLS	A549 Cells
Cell or tissue culture	Air–liquid interface (ALI)		
Exposure mode	Small volume liquid application on the apical surface	Aerosol exposures	Aerosol and vapor exposures on the apical surface
	Tissues were incubated with the test materials until the time point for sample collection.		
Treatment	Acetic acid (CAS 64-19-7), ammonium hydroxide (CAS 1336-21-6), benzyl isocyanate (CAS 3173-56-6), capsaicin (CAS 404-86-4), formaldehyde (CAS 50-00-0), toluene (CAS 108-88-3)		Acetic acid (CAS 64-19-7), ammonium hydroxide (CAS 1336-21-6), trimellitic anhydride (TMA, CAS 552-30-7), capsaicin (CAS 404-86-4), formaldehyde (CAS 50-00-0), toluene (CAS 108-88-3)
	Positive control: Triton™ X-100 (CAS 9002-93-1) and salicylic acid (CAS 69-72-7)	Positive control: sodium dodecyl sulfate (SDS, CAS 151-21-3) and salicylic acid (CAS 69-72-7)	
	Negative control: lactose (CAS 63-42-3)		
Endpoints	Time of sample collection: 1, 6, and 24 h		Time of sample collection: 6, and 24 h
	Viability (LDH release), resazurin metabolism *, barrier integrity (TEER), cytokine, oxidative stress	Viability (WST-8), cytokine, oxidative stress	Viability (WST-1), cytokine, oxidative stress, MMP, unspecified cell stress

* Resazurin metabolism was used for evaluating the effect on cell viability from formaldehyde exposures. hPCLS—human precision-cut lung slices, LDH—lactate dehydrogenase, TEER—trans-epithelial electrical resistance, MMP—mitochondrial membrane potential, WST-8—water soluble tetrazolium 8, and WST-1—water soluble tetrazolium 1.

**Table 2:** Physicochemical properties of the seven test chemicals used for droplet and/or vapor MPPD simulations.

Physicochemical Properties *	Units	Acetic acid (Aerosol/Vapor)	Ammonium Hydroxide (Aerosol/Vapor)	Benzyl Isocyanate (Aerosol)	Trimellitic Anhydride (Aerosol)	Capsaicin (Aerosol)	Formaldehyde (Aerosol)	FORMALDEHYDE (Vapor ^¥^)	Toluene (Aerosol/Vapor)
Particle properties									
Density	g/cm^2^	1.05	0.9	1.078	1.7	1	0.7		0.9
Diameter	µm	Multiple particles, size range 0.001 to 10							Multiple particles, size range 0.001 to 10
Compound exposure		Custom/high vapor pressure	Custom/high vapor pressure	Low vapor pressure	Low vapor pressure	Low vapor pressure	Custom		Custom/high vapor pressure
Diffusion coefficient in air **	cm^2^/s	0.113	0.154	0.0679	0.00000653	0.0418	0.177		0.0803
Molecular weight	g/mol	60.05	35.04	133.147	192.125	305.412	30.026		92.138
Saturated vapor pressure ^+^	g/cm/s^2^	146.6	0	5.34	0	0	46253		369.4
Surface tension	g/s^2^	31.9	72	37.8	79.4	35	12.6		28.8
Vapor properties									
Diffusion coefficient in air **	cm^2^/s	0.113	0.154				0.177	0.15	0.0803
Diffusion coefficient in tissue **	cm^2^/s	0.0000126	1.82 × 10^−5^				0.0000206	0.0000174	0.00000849
Diffusion coefficient in tissue **	cm^2^/s	0.0000126	1.82 × 10^−5^				0.0000206	0.0000174	0.00000849
Partition coefficient ^#^		44642.9	7077				263.16	72464	4.12
K_f_	1/s	0.1	0.1				0.1	0.018	0.1
K_m_	mg/m^3^	1	1				1	201	1
V_max_	mg/m^3^/s	0	0				0	7.86	0

* The physicochemical properties were obtained from either ChemSpider (https://www.chemspider.com/, accessed on 20 June 2024) or EPI Suite™ version 4.1 ** These values were obtained from the EPA On-line Tools for Site Assessment Calculation https://www3.epa.gov/ceampubl/learn2model/part-two/onsite/estdiffusion-ext.html (accessed on 20 June 2024). + Vapor pressure values were used instead of the saturated vapor pressure values. ^¥^ Measured values were used for formaldehyde vapor exposure simulation. # Partition coefficient was calculated as follows: Henry’s law constant (bond method) in Pa-m^3^/mol is divided by 298K and 8.314 gas constant. The resultant value is inversed to obtain the dimensionless partition coefficient.

**Table 3:** Effect of direct droplet liquid exposures to respiratory irritants in MucilAir™ cells. (**A**) Changes in LDH, TEER measurements, IL-6 release, and IL-8 release from acetic acid, ammonium hydroxide, benzyl isocyanate, capsaicin, and toluene exposures. (**B**) Changes in resazurin metabolism, TEER measurements, IL-6 release, and IL-8 release from formaldehyde exposures. The increasing orange color gradient shows increased cytotoxicity, whereas the increasing blue color gradient indicates increased cytokine release compared to time-matched controls. All treatments were conducted across a concentration range at 1, 6, and 24 h timepoints. Capsaicin was dissolved in Vehicle 2. Vehicle 1—DMSO 1%, saline 1% in dH_2_O. Vehicle 2—saline 1%, 0.16 mg/mL Pluronic^®^ F-127 in dH_2_O. Data were not analyzed for statistical significance due to single donor tissues with the n = 2/group. Complete data are available in the Supplementary Tables S1–S4. LDH—lactate dehydrogenase, TEER—trans-epithelial electrical resistance, and IL—interleukin.

(**A**)												
	**% Viable (100-** **% LDH Release)**			**% Control TEER**			**Relative Change IL-6 Release**			**Relative Change IL-8 Release**		
	**1 h**	**6 h**	**24 h**	**1 h**	**6 h**	**24 h**	**1 h**	**6 h**	**24 h**	**1 h**	**6 h**	**24 h**
Vehicle 1 (0 µg/cm^2^)	99.2	99.6	99.3	99.9	99.9	99.9	2.4	−0.2	0.3	1.1	0.8	0.3
Vehicle 2 (0 µg/cm^2^)	99.7	100.0	100.0	100.4	100.0	100.0	−0.5	−0.2	0.1	3.1	0.8	0.8
Lactose (91 µg/cm^2^)	99.9	99.9	99.9	277.0	134.5	113.7	−0.6	−0.1	0.7	−0.4	−0.5	−0.2
Salicylic acid (227 µg/cm^2^)	99.6	99.3	98.1	17.2	11.2	14.3	0.8	0.7	2.9	0.8	0.4	1.3
SDS (67 µg/cm^2^)	99.9	100.0	96.7	31.6	14.6	10.1	−0.2	1.2	2.8	1.5	1.4	1.3
Acetic acid (136 µg/cm^2^)	95.7	92.6	89.6	18.9	10.3	18.5	−0.1	1.3	1.4	1.6	0.8	0.5
Acetic acid (182 µg/cm^2^)	97.3	96.3	90.9	13.0	10.4	9.7	−0.4 h	0.4	2.1	0.6	0.4	0.8
Acetic acid (273 µg/cm^2^)	97.9	98.7	96.4	12.4	7.4	6.4	−0.6	−0.3	2.6	0.4	−0.3	1.1
Ammonium hydroxide (30 µg/cm^2^)	99.6	100.0	99.9	42.3	58.7	71.6	−0.1	−0.3	−0.3	0.8	0.0	1.3
Ammonium hydroxide (61 µg/cm^2^)	96.0	99.5	96.2	18.2	30.3	28.4	−0.4	0.0	0.2	0.8	0.3	0.7
Ammonium hydroxide (91 µg/cm^2^)	97.6	94.9	96.2	18.8	19.3	12.1	−0.1h	1.3	1.7	1.7	1.5	1.3
Benzyl isocyanate (91 µg/cm^2^)	97.9	90.6	78.4	26.1	10.0	10.7	0.4	0.8	1.7	0.3	0.2	0.5
Benzyl isocyanate (182 µg/cm^2^)	99.0	97.2	83.9	23.5	10.4	8.6	−0.7	0.0	3.6	−0.5	−0.4	0.9
Benzyl isocyanate (273 µg/cm^2^)	99.6	98.5	94.7	18.0	8.3	8.0	−0.7	0.4	1.1	−0.1	−0.7	−0.2
Capsaicin (9 µg/cm^2^)	100.0	100.0	99.9	94.3	41.5	57.5	0.5	0.8	0.6	0.9	1.5	0.6
Capsaicin (23 µg/cm^2^)	99.9	100.0	99.9	19.0	21.9	51.6	1.0	2.6	0.8	−0.8	1.6	0.6
Capsaicin (46 µg/cm^2^)	99.9	97.6	92.2	17.6	12.1	72.0	3.1	1.1	1.0	0.1	1.8	0.7
Toluene (136 µg/cm^2^)	98.9	99.4	99.3	112.9	108.7	82.7	−0.5	2.2	0.3	0.3	−0.1	1.3
Toluene (182 µg/cm^2^)	99.9	100.0	99.7	80.1	92.6	107.0	−0.3	0.7	2.0	0.3	0.0	1.3
Toluene (273 µg/cm^2^)	100.0	100.0	100.0	49.7	57.5	100.1	−0.6	−0.3	−0.4	0.2	0.2	1.3
(**B**)												
	**% Resazurin Metabolism**			**% Control TEER**			**Relative Change IL−6 Release**			**Relative Change IL−8 Release**		
	**1 h**	**6 h**	**24 h**	**1 h**	**6 h**	**24 h**	**1 h**	**6 h**	**24 h**	**1 h**	**6 h**	**24 h**
Vehicle 1 (0 µg/cm^2^)	100.0	100.0	100.0	99.9	99.9	99.9	2.4	−0.2	0.3	1.1	0.8	0.3
Lactose (91 µg/cm^2^)	72.2	88.9	151.3	277.0	134.5	113.7	−0.6	−0.1	0.7	−0.4	−0.5	−0.2
SDS (67 µg/cm^2^)	100.9	78.4	196.6	31.6	14.6	10.1	−0.2	1.2	2.8	1.5	1.4	1.3
Formaldehyde (9 µg/cm^2^)	71.0	119.6	57.1	50.0	66.8	77.7	−0.3	0.1	0.1	0.7	0.1	0.1
Formaldehyde (91 µg/cm^2^)	64.5	101.6	0.0	30.0	8.1	6.3	−0.3	0.2	−0.2	0.9	0.1	0.3
Formaldehyde (909 µg/cm^2^)	54.0	52.0	18.7	22.2	5.8	7.5	−0.9	hh−0.9	−0.8	−0.9	−1.0	−1.0
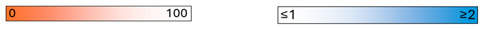												

**Table 4:** Effect of aerosol exposures to known irritants on viability and IL-6 and IL-8 releases across a concentration range at 1, 6, and 24 h timepoints in hPCLS tissues.

	% Control WST8—Viability			Relative Change IL-6 Release			Relative Change IL-8 Release		
	1 h	6 h	24 h	1 h	6 h	24 h	1 h	6 h	24 h
Vehicle 1 (0 µg/cm^2^)	100.0	100.0	100.0	1.1	−0.3	3.7	3.7	0.2	7.5
Lactose (526 µg/cm^2^)	100.0	96.9	79.5 *	−0.8	0.1	−0.6	−0.9	−0.3	−0.8
Salicylic acid (526 µg/cm^2^)	0.7 *	1.9 *	0.4 *	1.4	1.2	−0.6	4.9	6.4	0.3
Triton™ X−100 (263 µg/cm^2^)	0.1 *	0.9 *	0.5 *	1.2	1.0	−0.4	2.8	8.1	0.7
Acetic acid (26 µg/cm^2^)	92.0	97.6	92.5	−0.9	−0.h4	−0.7	−0.9	−0.7	−0.8
Acetic acid (263 µg/cm^2^)	56.7 *	44.3 *	14.7 *	−0.7	−0.9	−0.7	−0.7	−0.8	−0.7
Acetic acid (789 µg/cm^2^)	2.4 *	1.9 *	1.8 *	−0.8	−0.8	−1.0	−0.7	−0.6	−0.9
Ammonium hydroxide (789 µg/cm^2^)	0.5 *	2.2 *	1.0 *	−0.7	−0.8	−0.9	−0.7	−0.7	−0.8
Ammonium hydroxide (1316 µg/cm^2^)	0.4 *	0.7 *	0.3 *	−0.3	−0.6	−0.9	−0.3	−0.5	−0.8
Ammonium hydroxide (1842 µg/cm^2^)	0.4 *	0.6 *	0.3 *	−0.6	0.0	−0.8	−0.4	0.0	−0.8
Benzyl isocyanate (18 µg/cm^2^)	85.1 *	91.6	101.9	0.6	9.2 *	32.8 *	0.7	5.2	0.0
Benzyl isocyanate (184 µg/cm^2^)	42.0 *	25.9 *	32.7 *	0.5	0.5	−0.3	0.2	0.0	0.5
Benzyl isocyanate (1842 µg/cm^2^)	1.7 *	0.2 *	2.4 *	−0.6	−0.9	−1.0	−0.8	−0.9	−1.0
Capsaicin (11 µg/cm^2^)	86.4 *	90.1 *	95.1	1.1	0.6	9.0 *	0.9	1.5	7.7
Capsaicin (32 µg/cm^2^)	84.4	66.4 *	22.5 *	1.9	2.6	3.5	2.4	4.3	4.4
Capsaicin (95 µg/cm^2^)	35.1 *	16.9 *	0.5 *	0.0	2.0	−0.5	1.9	8.3	1.5
Formaldehyde (263 µg/cm^2^)	24.4 *	8.5 *	0.8 *	−0.5	−0.8	−0.8	−0.8	−0.8	−0.9
Formaldehyde (789 µg/cm^2^)	3.7 *	0.0 *	0.4 *	−0.6	−0.8	−1.0	−0.9	−1.0	−1.0
Formaldehyde (1842 µg/cm^2^)	0.3 *	0.0 *	0.3 *	−0.8	−0.9	−1.0	−1.0	−1.0	0.0
Toluene (26 µg/cm^2^)	102.0	93.2	39.7	0.2	−0.4	−0.9	−0.6	−0.5	−1.0
Toluene (263 µg/cm^2^)	100.4	104.7	90.4	−0.2	0.2	6.3 *	−0.6	1.2	9.7 *
Toluene (1842 µg/cm^2^)	95.0	105.6	94.1	−0.3	6.2 *	5.0 *	−0.6	6.0 *	5.1 *
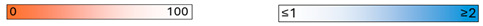									

The increasing orange color gradient shows an increase in cytotoxicity, whereas the increasing blue color gradient indicates an increase in cytokine release compared to time-matched controls. The effect on viability was analyzed for statistical significance using a student’s t-test; N = 6/group. Relative changes in IL-6 and IL-8 were analyzed using 2-way ANOVA with Dunnett’s multiple comparison test; N = 3/group. * *p* < 0.05 compared to time-matched vehicle control group. Complete data are available in the Supplementary Table S5.

**Table 5:** Effect of aerosol and vapor exposures to known irritants in A549 cell line cultures.

Treatment		Cytotoxicity (WST-1)		Mitochondrial Membrane Potential (MMP)	Unspecified Cell Stress
Substance	Exposure form	LC_50_ at 24 h	LOAEL at 24 h	LOAEL at 24 h	LOAEL at 24 h
Lactose	Aerosol (µg/cm^2^)	290.87	290.87	290.87	290.87
SDS	Aerosol (µg/cm^2^)	6.83	0.65	4.97	0.22
Acetic acid	Aerosol (µg/cm^2^)	41.7	9.21	16.04	11.7
	Vapor (ppm)	1016	787	501	295
Ammonium hydroxide	Aerosol (µg/cm^2^)	4.2	1.28	2.83	1.16
	Vapor (ppm)	571	323	157	76.9
Capsaicin	Aerosol (µg/cm^2^)	0.94	15.02	36.29	0.02
Trimellitic anhydride	Aerosol (µg/cm^2^)	84.36	5.88	5.02	0.42
Formaldehyde	Aerosol (µg/cm^2^)	29.95	5.82	3.45	3.45
	Vapor (ppm)	41	15	12	9
Toluene	Vapor (ppm)	20763	18862	26557	16006
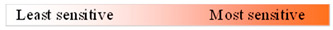					

The color gradient highlights concentrations showing the most sensitive (orange) endpoint to the least sensitive (white) endpoint measured. Results were analyzed by generating dose–response curves after calculating % of control values (test aerosol versus clean air control exposure). Dose–response curves were calculated by applying the best-fit strategy with a calculation of confidence levels at 95% using statistical software (Origin 2021b, OriginLab Corporation, Northampton, MA, USA). Calculated LC50-values for WST were defined by the concentration with an effect observed at 50% of the control value. The lowest dose showing significant differences in effects from the controls was considered the lowest observed adverse effect level (LOAEL). Complete data are available in Supplementary Figures S1 and S2.

**Table 6:** Summarizing the PODs based on irritant exposure effects presented in Table 4 and Table 5.

Test Compounds	hPCLS			A549	
	PODs (µg/cm^2^)	Exposure Duration	Associated Endpoint or Biomarker	LOAEL (µg/cm^2^)	Associated Endpoint or Biomarker
Acetic acid	263	1 h	Viability	9.21 (aerosol); 103.8 (vapor)	Viability; MMP
Ammonium hydroxide	NA	NA	Lethal at all concentrations and time points	1.28 (aerosol); 99.6 (vapor)	Viability; MMP
Benzyl isocyanate	18	6 h	IL-6 and IL-8 release	NA	NA
Trimellitic anhydride	NA	NA	NA	5.02	MMP
Capsaicin	32	6 h	Viability and IL-6/IL-8 release	15.02	Viability
Formaldehyde	NA	NA	Lethal at all concentrations and time points	3.45 (aerosol); 17.4 (vapor)	MMP
Toluene	263	24 h	IL-6 and IL-8 release	7476 (vapor)	Viability

NA = not applicable; MMP = mitochondrial membrane potential.

**Table 7:** Comparison of simulated human-relevant exposures with experimental in vitro exposures.

Test Compounds	MPPD Simulation Using CCIII Inhalation TTC as Exposure Concentration	hPCLS POD	A549 LOAEL	* Margin of Exposure—Aerosol (MOE_a_)	Margin of Exposure—Vapor (MOE_v_)
	Deposition After 1 h Exposure (µg/cm^2^)	Exposure (µg/cm^2^)	Exposure (µg/cm^2^)		
Acetic acid	0.009 (aerosol); 0.0031 (vapor)	263 (1 h)	9.21 (aerosol); 103.8 (vapor)	1023	11533
Ammonium hydroxide	0.0096 (aerosol); 0.0035 (vapor)	NA	1.28 (aerosol); 99.6 (vapor)	133	28457
Benzyl isocyanate	0.001	18 (6 h)	NA	18000	NA
Trimellitic anhydride	0.001	NA	5.02	5020	NA
Capsaicin	0.001	32 (6 h)	15.02	15020	NA
Formaldehyde	0.019 (aerosol); 0.043 (vapor)	NA	3.45 (aerosol); 17.4 (vapor)	181.6	404.7
Toluene	0.007 (aerosol); 0.022 (vapor)	263 (24 h)	7476 (vapor)	37571	339818

* MOE for aerosol exposures was calculated using the lowest observed experimental concentration between hPCLS and A549 cell model. NA—not applicable.

## Data Availability

The original contributions presented in the study are included in the article/Supplementary Material, further inquiries can be directed to the corresponding author.

## Supplementary Materials

The following supporting information can be downloaded at: https://www.mdpi.com/article/10.3390/toxics13010035/s1, Table S1. Effect on LDH release due to direct droplet liquid exposures of irritants in MucilAir™ tissues at (a) 1 h, (b) 6 h, and (c) 24 h exposure; Table S2. Changes in Resazurin metabolism due to formaldehyde exposures in MucilAir™; Table S3. Changes in Trans Epithelial Electrical Resistance (TEER) due to irritant exposures in MucilAir™; Table S4. Changes in cytokine release due to irritant exposures in MucilAir™; Table S5. Changes in viability and cytokine release in hPCLS due to irritant exposures; Figure S1. Dose-response curves for viability and IL-8 release at 24 h in A549 cells. (a) Lactose, aerosol, (b) Salicylic acid, (c) SDS—dry particle aerosol, (d) ammonium hydroxide—aerosol, (e) acetic acid—aerosol, (f) formaldehyde—aerosol, (g) trimellitic anhydride—dry particle aerosol, (h) capsaicin—aerosol, (i) ammonium hydroxide—vapor, (j) acetic acid—vapor, (k) formaldehyde—vapor, (l) toluene—vapor; Figure S2. Dose-response curves for mitochondrial membrane potential (MMP) and unspecified cellular stress measurements at 24 h in A549 cells. (a) Lactose, aerosol, (b) Salicylic acid, (c) SDS—dry particle aerosol, (d) ammonium hydroxide—aerosol, (e) acetic acid—aerosol, (f) formaldehyde—aerosol, (g) trimellitic anhydride—dry particle aerosol, (h) capsaicin—aerosol, (i) ammonium hydroxide—vapor, (j) acetic acid—vapor, (k) formaldehyde—vapor, (l) toluene—vapor.

## Appendix A

Highlighted below are specific procedures employed in the study design using six known respiratory irritants blind tested in three in vitro models.

### Appendix A.1. MucilAir™ Model

#### Appendix A.1.1. Cell Culture

This work utilized the ‘single donor’ variant constructed from the nasal epithelial cells of a single, healthy donor. MucilAir™ tissues delivered from the supplier were transferred to 24-well plates containing MucilAir™ medium (700 µL). The tissues were allowed to recover in an incubator (37 °C temperature with a 5% CO_2_) for 1–2 week(s) with media being replaced every 2–3 days before exposure. All incubations of cells were performed in humidified incubators set to maintain a temperature of 37 °C with a 5% CO_2_ atmosphere (standard culture conditions). TEER was measured 3 days prior to dosing using a MilliCell ERS-2 m and chopstick electrodes to allow sufficient time for the mucus layer to reform before exposure to the chemicals. Only tissues with TEER >200 Ω × cm^2^ were included in the experiment.

#### Appendix A.1.2. Test Chemical Exposure

Each treatment was applied to 6 MucilAir™ tissues, with the exception of LDH control treatments which were applied to 4 tissues. The dosing volume for all treatments was 10 μL with the exception of salicylic acid which was applied as a 30 μL application (due to solubility limit). The test chemical solutions were applied evenly using a pipette tip and by gentle tapping of the plate. An additional set of 6 MucilAir™ tissues were untreated and served as ALI controls. The concentrations were converted from µg/mL to µg/cm^2^ using 0.33 cm^2^ surface area of the MucilAir™ tissues cultured in 24-well plates (Table A1).

**Table A1:** Summary of test chemical concentrations in a water-based vehicle.

Chemical	Concentrations (µg/mL)	Concentrations Converted to µg/ cm^2^	Vehicle
Acetic Acid	9000, 6000, 4500	136, 182, 273	Ultrapure water containing 1% DMSO, 1% Physiological saline
Ammonium Hydroxide	3000, 2000, 1000	30, 61, 91	
Benzyl Isocyanate	9000, 6000, 3000	91, 182, 273	
Toluene	300, 3000, 30000	136, 182, 273	
Formaldehyde	4500, 6000, 9000	9, 91, 909	
Capsaicin	300, 750, 1500	9, 23, 46	Ultrapure water containing 1% physiological saline and 0.16 mg/mL Pluronic^®^ F-127

As aerosol applications were not feasible with the available equipment, a 10 μL volume was used for a majority of the treatments to mimic an air–liquid application as closely as possible using a pipette. Tissues were incubated under standard culture conditions for their respective exposure period.

#### Appendix A.1.3. Endpoint Evaluation

At 1, 6, and 24 h post-exposure, 2 replicate tissues from each group were collected for endpoint evaluations.

Liquid which permeated into the apical chamber was collected and combined with the basal media and retained. The tissue surface was then rinsed with saline (3 × 0.5 mL) and then tissues were processed for toxicity evaluation by TEER, resazurin assay (Formaldehyde and appropriate controls only), and histopathology evaluation. Samples of spent culture media were collected for evaluation of LDH release (CytoToxONE, Promega, all chemicals except Formaldehyde), and release of inflammatory markers (IL-8, IL-6, GM-CSF, MCP-1, TNF-α, and IL-1β) by Luminex and generation of reactive oxygen species (ROS) using an Acridan Lumigen Reagent as described by [40].

### Appendix A.2. Human Precision-Cut Lung Slice (hPCLS) Model

#### Appendix A.2.1. hPCLS Preparation and Culture

A non-transplantable non-diseased human lung was obtained via the National Disease Research Interchange (NDRI; Philadelphia, PA, USA). The donor was a non-smoker 67 years old Caucasian female (height: 150 cm; weight: 54.4 Kg) and the lung was procured through the Organ Procurement Organizations using the United Network for Organ Sharing (UNOS) identification guidelines, with authorization obtained from the donor’s next of kin and maintaining patient confidentiality.

Upon receipt, the lung was visually inspected to ascertain the quality and usable tissue (free of gross lesions or other abnormalities that would preclude use) before coring and slicing. The lung slices were obtained by following the process outlined by Patel et al. [25] and maintained at standard culture conditions (SCC; i.e., 37 ± 1 °C, 5% CO_2_, and 90% relative humidity) throughout the culture period.

#### Appendix A.2.2. Aerosol Exposures

The test chemical(s) and controls (except lactose) were dissolved directly in dimethyl sulfoxide (DMSO; Millipore Sigma, St. Louis, MO, USA) to prepare dosing solutions at concentrations indicated in Table A2. The lactose dosing solution was prepared in 5% DMSO in DMEM:F-12 (1:1) medium. The pH of the dosing solutions was measured using MQuant^®^ pH-indicator strips (Millipore Sigma, St. Louis, MO, USA).

**Table A2:** Summary of exposure concentrations and pH measurements of the dosing solutions.

Treatment	Nebulization for Aerosol Exposure		Estimated Deposition	pH
	Concentration (µg/mL)	Volume (mL)	(µg/cm^2^)	
Air	NA	NA	NA	NA
Vehicle (DMSO)	0	0.0	0	8.7
Lactose *	11,899	0.05	526	8.0
Triton™ X-100	119,763	0.5	263	8.5
Salicylic acid	239,526	0.5	526	5.0
Acetic acid	11,976	0.5	26	7.0
	119,763	0.5	263	4.5
	359,289	0.5	789	5.0
Ammonium hydroxide	359,289	0.5	789	12.5
	598,816	0.5	1316	12.0
	838,342	0.5	1842	12.0
Benzyl isocyanate	8383	0.5	18	9.0
	83,834	0.5	184	9.0
	838,342	0.5	1842	7.0
Capsaicin	4791	0.5	11	9.0
	14,372	0.5	32	9.0
	43,115	0.5	95	10.0
Formaldehyde	119,763	0.5	263	8.0
	359,289	0.5	789	5.0
	838,342	0.5	1842	4.0
Toluene	11,976	0.5	26	10.0
	119,763	0.5	263	8.0
	838,342	0.5	1842	5.0

* Delivered on the apical surface of the tissues using a pipette; NA = not applicable.

For aerosol exposures, the insert holders on the Vitrocell^®^ Cloud 12 base module (culture surface area of 1.12 cm^2^) were filled with approximately 3.1 mL of Hanks’ Balanced Salt Solution (HBSS). Tissue inserts were placed into the insert holders of the exposure chamber (contained in a biosafety cabinet). Five hundred microliters of the test chemicals or control dosing solution/suspension were placed into the Vitrocell^®^ Cloud 12 nebulizer reservoir and spiked with 5 µL of ~0.011% saline solution to ascertain adequate nebulization. The nebulizer was activated until the entire volume of the solution/suspension was discharged into the exposure chamber. Ten to 15 min after deposition of the aerosol, the tissue inserts were removed from the chamber and returned to standard culture conditions (SCC) until harvest at 1, 6, or 24 h. The aerosol mass depositions were theoretically calculated assuming 60% deposition efficiency using the following equation.

(A1) $$\begin{array}{l} Deposited\;mass\;(\frac{\mu g}{{cm}^{2}})\\ =\frac{Concentration\;\left(\frac{\mu g}{mL}\right)\times Nebulized\;volume\;\left(mL\right)\times Deposition\;factor}{Base\;surface\;area\;({\mathrm{cm}}^{2})} \end{array}$$

where base surface area = 136.53 cm^2^ (Cloud exposure chamber), nebulized volume = 0.5 mL, and deposition factor = 0.6 (i.e., 60% efficiency).

#### Appendix A.2.3. Endpoint Evaluation

##### hPCLS Viability—WST-8 Assay

The hPCLS viability was assessed using WST-8 assay by following the procedure outlined as per Patel et al. [25]. The percent viability was calculated as follows, using background (blank) subtracted values:

(A2) $$\%Viability=\frac{{OD}_{450}\;of\;Test\;Chemical}{{OD}_{450}\;of\;Vehicle\;Control}\times 100.$$

##### Cytokine/Chemokine Analysis

Culture medium samples from the hPCLS were collected and assessed for TNFα, IL-1β, IL-6, IL-8, MCP-1, and GM-CSF using a Luminex assay kit (R&D Systems, Minneapolis, MN, USA). The samples were run on a Luminex MAGPIX system (Luminex, Austin, TX, USA) and the data were analyzed using the Luminex xPONENT^®^ software version 4.3.

##### Oxidative Stress Analysis

The ratio of reduced glutathione (GSH) to oxidized glutathione (GSSG) was used as a marker of oxidative stress. After the viability assessment, the hPCLS (n = 3/group) was rinsed with HBSS and lysed in 5% sulfosalicylic acid (Millipore Sigma, St. Louis, MO, USA) using TissueLyser II (Qiagen, Germantown, MD, USA). The lysates were centrifuged at 10,000× *g*, 4 °C for 10 min. The lysate supernatants were stored at ≤−60 °C until the assessment of GSH and GSSG using GSH/GSSG-Glo™ assay kit (Promega, Madison, WI, USA). The background corrected relative light units (RLU) that reflect the amount of total GSH and GSSG measured in the assay were used to calculate the GSH/GSSG ratio as follows:

(A3) $$Ratio\;GSH/GSSH=\frac{\left(Net\;total\;GSH\;RLU-Net\;GSSG\;RLU\right)}{\left(Net\;GSSG\;RLU÷2\right)}$$

#### Appendix A.2.4. Statistical Analyses

The data are presented as the mean ± standard deviation (SD) and statistically compared using student’s t-test (2-tailed, 2-sample unequal variance; Microsoft Excel) or two-way ANOVA with Dunnett’s multiple comparisons test (GraphPad Prism). A *p* < 0.05 was considered statistically significant compared to respective vehicle control groups at indicated time points. Refer to Supplement Table S5.

### Appendix A.3. A549 Cell Model

#### Appendix A.3.1. Cell Culture

Cells were routinely taken from a stock pool and grown in 75 cm^2^ flasks by use of Dulbecco’s MEM medium (Seromed, Berlin, Germany) supplemented with 10% fetal bovine serum (FBS) and antibiotics (0.01% Gentamicin) at 37 °C in a humidified atmosphere containing 5% CO_2_. Cells were passed every 3–4 days. During each passage, microscopic observation was conducted, and cell quality and quantity were checked using an electronic cell counter (CASY^®^ Cell Counter + Analyzer System; Schärfe System, Reutlingen, Germany). During the cell passage, an aliquot of the cells was seeded on microporous membranes (0.4 µL per 1 cm^2^; BD Falcon). Cells were cultivated on the membranes for approximately 72 h until they reached a confluent monolayer as inspected by light microscopy. Eighteen hours before exposure residual liquid was removed from the apical side of the layer and the culture medium in the basolateral compartment was replaced by the serum-free medium. Individual cell exposure of cultures was conducted under ALI conditions using an optimized exposure setup (P.R.I.T.^®^ ExpoCube^®^) in 12-well plates [41].

#### Appendix A.3.2. Test Chemical Exposure

Gaseous test atmospheres were generated by evaporation of liquids using a syringe-based test gas generator (Gasmet) and Fourier transform infrared spectroscopy (FT-IR, Gasmet DX4000) for online analysis of the vapor concentrations during cell exposures. Aerosols were generated using dilutions of the chemical in water, acetone, or butanal. For organic solvents, aerosols were dried in clean air to evaporate the organic solvent to the gas phase. Aerosol concentrations were followed by light scattering (custom photometer, Fh-ITEM) and gravimetrical filter analysis.

Cultures from single plates were exposed to the test vapors/aerosols, to clean air as exposure control, or were non-exposed (no exposure flow) in three experimental groups at the same time. Exposure conditions included the range between 2 and 10 mL per min per cm^2^ culture surface and an exposure time of up to 60 min, dependent on the applied dose. Vapor phase exposures were performed for 60 min for all test chemicals tested as vapor.

#### Appendix A.3.3. Endpoint Evaluation

In vitro read-outs included analysis of viability (WST-1), unspecific cellular stress (Hoechst 33258 staining), mitochondrial membrane potential (MMP) using JC-1 staining, and cytokine release [42]. Briefly, viability was checked using the WST-1 assay at 24 h, cellular morphology was assessed by microscopic inspection at 6 and 24 h, and Hoechst and JC-1 staining was analyzed at 0, 6, and 24 h. The mitochondrial membrane potential dye JC-1 was obtained from molecular probes, while Hoechst 33258 was purchased from Sigma Aldrich. Image analysis of the life fluorescence staining was achieved using an Olympus ScanR-software package (ScanR Analysis version 3.01) in a custom image analysis process. Cytokine secretion (IL-8, IL-1β, GM-CSF, MCP-1) into basal media was analyzed using commercial ELISA kits (R & D Systems) at 6 and 24 h after exposure.

#### Appendix A.3.4. Statistical Analysis

Results were analyzed by generating dose–response curves after calculating % of control values (test aerosol versus clean air control exposure). Dose–response curves were calculated by applying the best-fit strategy with a calculation of confidence levels at 95% using statistical software (Origin 2021b, Originlab Corporation, Northampton, MA, USA). Calculated LC_50_-values for WST were defined by the concentration with an effect observed at 50% of the control value. The lowest dose showing significant differences in effects from the controls was considered the lowest observed adverse effect level (LOAEL).
