# Supplementary material for: A Proof-of-Concept for Safety Evaluation of Inhalation Exposure to Known Respiratory Irritants Using In Vitro and In Silico Methods
Source: Toxics. 2025 Jan 4;13(1):35. doi: 10.3390/toxics13010035 (PMC11769436; doi:10.3390/toxics13010035)
Supplement: Supplementary file 1 [file toxics-13-00035-s001.zip › toxics-3315840-supplementary.pdf]

Supplement: Background Data

1) MucilAir™

Table S1. Effect on LDH release due to direct droplet liquid exposures of irritants in MucilAir™ tissues.

Table S1a. 1 hour exposure

Treatment	LDH Release (%)	LDH Release Mean (%)
**LDH Max**	92.76	100.00
	112.71	
	93.02	
	101.50	
**VC 1 (DMSO 1%, Saline 1% in ultrapure water)**	1.03	0.78
	0.53	
**VC 2 (1% Saline, 0.16 mg/mL Pluronic F-127 in ultrapure water)**	0.00	0.29
	0.59	
**NC (β-Lactose)**	0.16	0.08
	0.00	
**UT (Air Liquid Interface)**	0.11	0.05
	0.00	
**PC 1 (SDS)**	0.13	0.07
	0.00	
**PC 2 (Salicylic Acid)**	0.12	0.43
	0.73	
**PC 3 (Lipopolysaccharide)**	1.58	1.31
	1.03	
**Acetic acid (136 μg/cm²)**	3.92	4.32
	4.72	
**Acetic acid (182 μg/cm²)**	3.19	2.74
	2.30	
**Acetic acid (273 μg/cm²)**	2.31	2.10
	1.88	
**Ammonium hydroxide (30 μg/cm²)**	0.76	0.38
	0.00	
**Ammonium hydroxide (61 μg/cm²)**	3.74	4.05
	4.36	

**Ammonium hydroxide (91 μg/cm²)**	4.08	2.39
	0.70	
**Benzyl isocyanate (91 μg/cm²)**	2.18	2.06
	1.94	
**Benzyl isocyanate (182 μg/cm²)**	1.25	0.98
	0.71	
**Benzyl isocyanate (273 μg/cm²)**	0.36	0.38
	0.40	
**Capsaicin (9 μg/cm²)**	0.02	0.01
	0.00	
**Capsaicin (23 μg/cm²)**	0.28	0.14
	0.00	
**Capsaicin (46 μg/cm²)**	0.00	0.12
	0.24	
**Toluene (136 μg/cm²)**	1.32	1.06
	0.80	
**Toluene (182 μg/cm²)**	0.13	0.06
	0.00	
**Toluene (273 μg/cm²)**	0.03	0.04
	0.05	

Table S1b. 6 hours exposure

**Treatment**	**LDH Release (%)**	**LDH Release Mean (%)**
**LDH Max**	92.76	100.00
	112.71	
	93.02	
	101.50	
**VC 1 (DMSO 1%, Saline 1% in ultrapure water)**	0.09	0.37
	0.64	
**VC 2 (1% Saline, 0.16 mg/mL Pluronic F-127 in ultrapure water)**	0.00	0.00
	0.00	
**NC (β-Lactose)**	0.00	0.11

	0.22	
**UT (Air Liquid Interface)**	0.00	0.00
	0.00	
**PC 1 (SDS)**	0.00	0.00
	0.00	
**PC 2 (Salicylic Acid)**	1.50	0.75
	0.00	
**PC 3 (Lipopolysaccharide)**	0.20	0.42
	0.65	
**Acetic acid (136 µg/cm²)**	8.45	7.40
	6.35	
**Acetic acid (182 µg/cm²)**	3.69	3.69
	3.69	
**Acetic acid (273 µg/cm²)**	1.06	1.35
	1.65	
**Ammonium hydroxide (30 µg/cm²)**	0.00	0.00
	0.00	
**Ammonium hydroxide (61 µg/cm²)**	0.19	0.51
	0.83	
**Ammonium hydroxide (91 µg/cm²)**	5.26	5.13
	5.00	
**Benzyl isocyanate (91 µg/cm²)**	10.96	9.38
	7.79	
**Benzyl isocyanate (182 µg/cm²)**	1.55	2.85
	4.16	
**Benzyl isocyanate (273 µg/cm²)**	1.01	1.51
	2.01	
**Capsaicin (9 µg/cm²)**	0.00	0.01
	0.02	
**Capsaicin (23 µg/cm²)**	0.00	0.00
	0.00	
**Capsaicin (46 µg/cm²)**	0.00	2.41
	4.82	
**Toluene (136 µg/cm²)**	0.30	0.64
	0.99	
	0.00	0.00

**Toluene (182 µg/cm²)**	0.00	
**Toluene (273 µg/cm²)**	0.00	0.00
	0.00	

Table S1c. 24 hours exposure

Treatment	LDH Release (%)	LDH Release Mean (%)
**LDH Max**	92.76	100.00
	112.71	
	93.02	
	101.50	
**VC 1 (DMSO 1%, Saline 1% in ultrapure water)**	0.71	0.74
	0.77	
**VC 2 (1% Saline, 0.16 mg/mL Pluronic F-127 in ultrapure water)**	0.00	0.03
	0.07	
**NC (β-Lactose)**	0.00	0.12
	0.23	
**UT (Air Liquid Interface)**	0.00	0.00
	0.00	
**PC 1 (SDS)**	3.92	3.25
	2.58	
**PC 2 (Salicylic Acid)**	1.61	1.93
	2.25	
**PC 3 (Lipopolysaccharide)**	0.57	0.58
	0.60	
**Acetic acid (136 µg/cm²)**	11.90	10.45
	8.99	
**Acetic acid (182 µg/cm²)**	7.37	9.11
	10.85	
**Acetic acid (273 µg/cm²)**	3.85	3.64
	3.42	

**Ammonium hydroxide (30 μg/cm²)**	0.24	0.12
	0.00	
**Ammonium hydroxide (61 μg/cm²)**	4.27	3.80
	3.33	
**Ammonium hydroxide (91 μg/cm²)**	9.91	11.65
	13.39	
**Benzyl isocyanate (91 μg/cm²)**	23.78	21.59
	19.39	
**Benzyl isocyanate (182 μg/cm²)**	13.12	16.13
	19.14	
**Benzyl isocyanate (273 μg/cm²)**	6.37	5.31
	4.25	
**Capsaicin (9 μg/cm²)***	0.12	0.06
	0.00	
**Capsaicin (23 μg/cm²)***	0.12	0.15
	0.17	
**Capsaicin (46 μg/cm²)***	15.48	7.83
	0.19	
**Toluene (136 μg/cm²)**	0.57	0.70
	0.82	
**Toluene (182 μg/cm²)**	0.55	0.27
	0.00	
**Toluene (273 μg/cm²)**	0.00	0.01
	0.02	

Changes in LDH release from MucilAir™ as a measure of cytotoxicity due to exposure to known irritants across a concentration range at (a) 1-, (b) 6-, and (c) 24-hour time points. *Test item dissolved in Vehicle 2. Vehicle 1 - DMSO 1%, Saline 1% in dH2O. Vehicle 2 - Saline 1%, 0.16 mg/ml Pluronic® F-127 in dH2O. No statistical analysis due to a single donor with 2 replicates per group.

Table S2. Changes in Resazurin metabolism due to formaldehyde exposures in MucilAir™.

Treatment	Timepoint					
	1 h		6 h		24 h	
	RLU Mean	Mean % of Vehicle Control	RLU Mean	Mean % of Vehicle Control	RLU Mean	Mean % of Vehicle Control
**UT (Air Liquid Interface)**	6.44	38.10	6.12	52.35	2.43	32.36
**VC 1 (DMSO 1%, Saline 1% in ultrapure water)**	16.9	100.0	11.7	100.0	7.5	100.0
**Lactose (91 μg/cm²)**	12.2	72.2	10.4	88.9	11.4	151.3
**SDS (67 μg/cm²)**	17.0	100.9	9.2	78.4	14.8	196.6
**Formaldehyde (9 μg/cm²)**	12.0	71.0	14.0	119.6	4.3	57.1
**Formaldehyde (91 μg/cm²)**	10.9	64.5	11.9	101.6	-1.3	0.0
**Formaldehyde (909 μg/cm²)**	9.1	54.0	6.1	52.0	1.4	18.7

All treatments were conducted across a concentration range at 1-, 6-, and 24-hour timepoints. Vehicle 1 - DMSO 1%, Saline 1% in dH2O. Data was not analyzed for statistical significance due to single donor tissues with 2 replicates per group.

Table S3. Changes in Trans Epithelial Electrical Resistance (TEER) due to irritant exposures in MucilAir™.

Treatment	Timepoint		
	Mean TEER (Ω x cm²)		
	1h	6h	24h
UT (ALI)	789	582	465
Vehicle 1 (0 μg/cm²)	219	446	575
Vehicle 2 (0 μg/cm²)	119	295	520
Lactose (91 μg/cm²)	607	600	653
Salicylic Acid, 2.5 mg/mL	38	50	82
SDS (67 μg/cm²)	69	65	58
Acetic acid (136 μg/cm²)	41	46	106

Acetic acid (182 µg/cm²)	29	46	56
Acetic acid (273 µg/cm²)	27	33	37
Ammonium hydroxide (30 µg/cm²)	93	262	411
Ammonium hydroxide (61 µg/cm²)	40	135	163
Ammonium hydroxide (91 µg/cm²)	41	86	69
Benzyl isocyanate (91 µg/cm²)	57	44	62
Benzyl isocyanate (182 µg/cm²)	51	46	50
Benzyl isocyanate (273 µg/cm²)	39	37	46
Capsaicin (9 µg/cm²)*	113	122	299
Capsaicin (23 µg/cm²)*	116	131	337
Capsaicin (46 µg/cm²)*	139	70	335
Formaldehyde (9 µg/cm²)	109	298	446
Formaldehyde (91 µg/cm²)	66	36	36
Formaldehyde (909 µg/cm²)	49	26	43
Toluene (136 µg/cm²)	247	485	475
Toluene (182 µg/cm²)	175	413	615
Toluene (273 µg/cm²)	109	256	575

* Test item dissolved in Vehicle 2. Vehicle 1 - DMSO 1%, Saline 1% in dH2O. Vehicle 2 - Saline 1%, 0.16 mg/ml Pluronic® F-127 in dH2O. No statistical analysis due to a single donor with 2 replicates per group.

Table S4. Changes in cytokine release due irritant exposures in MucilAir™.

**Test Article**	**Exposure duration**	**Liquid Application**	**IL-6 pg/ml**	**IL-8 pg/ml**
	**hours**	**(µg/cm²)**		
**UT (ALI)**	1	0	7.09	218
	6	0	88.3	1755
	24	0	91.5	3230
**Vehicle 1**	1	0	24.35	449
	6	0	73.7	3230
	24	0	116.85	4285

**Vehicle 2**	1	0	10.96	902
	6	0	55.7	3110
	24	0	126.2	5775
**Lactose**	1	91	10.38	256
	6	91	65.95	1685
	24	91	203	3620
**Salicylic acid**	1	227	44.85	815
	6	227	123.35	4525
	24	227	453	10000
**SDS**	1	67	18.8	1135
	6	67	162.5	7815
	24	67	449.5	10000
**Acetic Acid**	1-hr	136	22.65	1161
		182	14.965	709
		273	9.875	613
	6-hr	136	167.5	5860
		182	103.05	4425
		273	54.8	2250
	24-hr	136	275	6505
		182	363	7785
		273	417	9175
**Ammonium hydroxide**	1-hr	30	21.95	795
		61	14.6	799
		91	21.1	1190
	6-hr	30	51.75	3280
		61	77.35	4055
		91	168	8050
	24-hr	30	84	10000
		61	136.5	7265
		91	316.5	10000
**Benzyl isocyanate**	1-hr	91	34	597
		182	6.56	237
		273	7.87	389
	6-hr	91	133.25	3970
		182	77.05	2075
		273	106.2	922
	24-hr	91	310	6575
		182	534.5	8160

		273	240	3260
**Capsaicin***	1-hr	9	16.9	1710
		23	22.2	210
		46	45.025	963
	6-hr	9	99.65	7715
		23	200	8145
		46	115.95	8652
	24-hr	9	204.5	9230
		23	230.5	9400
		46	247	10000
**Formaldehyde**	1-hr	9	16.3	754
		91	16.7	835
		909	3.195	28.3
	6-hr	9	84.15	3600
		91	84.85	3670
		909	6.32	7.1
	24-hr	9	126	4705
		91	97.2	5625
		909	19.3	4.53
**Toluene**	1-hr	136	13	601
		182	17.7	565
		273	9.97	526
	6-hr	136	238.5	2890
		182	125.15	3260
		273	50.65	3745
	24-hr	136	150.5	10000
		182	348	10000
		273	74.4	10000

*Test item dissolved in Vehicle 2. Vehicle 1 - DMSO 1%, Saline 1% in dH2O. Vehicle 2 - Saline 1%, 0.16 mg/ml Pluronic® F-127 in dH2O. No statistical analysis due to a single donor with 2 replicates per group.

2) Human Precision-Cut Lung Slices

Table S5. Changes in viability and cytokine release in hPCLS due to irritant exposures.

Test Article	Exposure duration	Estimated Aerosol Deposition	Viability			IL-6 pg/mL			IL-8 pg/mL		
	hours	(µg/cm²)	Avg	±	SD	AVE	±	SD	AVE	±	SD
ALI control	1	NA	94.8	±	4.7	40	±	14	25	±	7
	6	NA	98.1	±	8.5	281	±	75	155	±	56
	24	NA	44.8	±	47.4	110	±	18	65	±	27
Vehicle	1	0	100	±	5.3	85	±	45	116	±	66
	6	0	100	±	8.2	196	±	51	193	±	95
	24	0	100	±	9.0	512	±	263	557	±	270
Lactose	1	526	100.0	±	3.3	21	±	6	12	±	4
	6	526	96.9	±	13.8	210	±	168	137	±	75
	24	526	79.5	±	5.1	214	±	106	130	±	27
Salicylic acid	1	526	0.7	±	0.3	205	±	29	681	±	222
	6	526	1.9	±	2.1	439	±	141	1419	±	312
	24	526	0.4	±	0.1	187	±	84	750	±	203
Triton™ X-100	1	263	0.1	±	0.1	186	±	95	443	±	118
	6	263	0.9	±	0.1	386	±	213	1749	±	1650
	24	263	0.5	±	0.1	295	±	121	952	±	674
Acetic Acid	1-hr	26	92.0	±	9.0	11	±	6	9	±	6
		263	56.7*	±	13.4	25	±	16	30	±	20
		789	2.4*	±	0.2	17	±	9	29	±	12
	6-hr	26	97.6	±	9.9	126	±	80	65	±	16
		263	44.3*	±	10.8	15	±	7	33	±	12
		789	1.9*	±	0.4	30	±	14	75	±	18
	24-hr	26	92.5	±	3.8	165	±	10	135	±	28
		263	14.7*	±	7.3	168	±	270	160	±	190
		789	1.8*	±	0.2	8	±	1	46	±	18
Ammonium hydroxide	1-hr	789	0.5*	±	0.1	25	±	8	39	±	8
		1316	0.4*	±	0.1	57	±	5	85	±	34
		1842	0.4*	±	0.1	35	±	19	72	±	6
	6-hr	789	2.2*	±	0.9	46	±	11	67	±	15
		1316	0.7*	±	0.1	70	±	36	98	±	37
		1842	0.6*	±	0.1	LBC	±	ND	LBC	±	ND
	24-hr	789	1.0*	±	0.7	70	±	18	121	±	68

		1316	0.3*	±	0.1	48	±	23	111	±	44
		1842	0.3*	±	0.1	94	±	29	113	±	33
**Benzyl isocyanate**	1-hr	18	85.1*	±	8.4	140	±	46	193	±	92
		184	42.0*	±	10.4	130	±	71	135	±	106
		1842	1.7*	±	0.7	36	±	0	23	±	9
	6-hr	18	91.6	±	8.1	1991*	±	501	1203	±	458
		184	25.9*	±	13.7	287	±	112	195	±	118
		1842	0.2*	±	0.3	15	±	5	10	±	5
	24-hr	18	101.9	±	8.1	17288*	±	ND	>	±	ND
		184	32.7*	±	8.8	344	±	56	858	±	237
		1842	2.4*	±	0.6	9	±	5	4	±	1
**Capsaicin**	1-hr	11	86.4*	±	5.0	178	±	63	217	±	91
		32	84.4	±	15.4	245	±	64	391	±	149
		95	35.1*	±	8.0	84	±	68	333	±	289
	6-hr	11	90.1*	±	5.3	315	±	55	478	±	190
		32	66.4*	±	13.5	707	±	425	1021	±	294
		95	16.9*	±	8.4	595	±	219	1802	±	484
	24-hr	11	95.1	±	5.1	5140*	±	4094	4835	±	989
		32	22.5*	±	12.3	2302	±	1726	3012	±	1584
		95	0.5*	±	0.1	259	±	84	1407	±	450
**Formal-dehyde**	1-hr	263	24.4*	±	2.9	42	±	20	26	±	4
		789	3.7*	±	1.5	31	±	10	12	±	2
		1842	0.3*	±	0.2	21	±	9	5	±	2
	6-hr	263	8.5*	±	3.7	44	±	20	37	±	25
		789	0.0*	±	0.1	35	±	14	6	±	5
		1842	0.0*	±	0.0	17	±	7	0	±	ND
	24-hr	263	0.8*	±	0.1	84	±	22	36	±	5
		789	0.4*	±	0.1	18	±	4	3	±	ND
		1842	0.3*	±	0.0	5	±	3	<	±	ND
**Toluene**	1-hr	26	102.0	±	9.5	98	±	31	51	±	14
		263	100.4	±	9.2	69	±	29	45	±	25
		1842	95.0	±	6.3	60	±	19	49	±	9
	6-hr	26	93.2	±	5.9	119	±	63	97	±	69
		263	104.7	±	8.1	236	±	79	423	±	180
		1842	105.6	±	6.5	1405*	±	1119	1353*	±	594
	24-hr	26	39.7	±	43.9	41	±	11	20	±	5
		263	90.4	±	8.5	3754*	±	1762	5959*	±	2705

		1842	94.1	±	7.5	3070*	±	540	3416*	±	1000
					**HSC**	993			953		
					**LSC**	4			4		

BC = Baseline; VC = Vehicle; Lac = Lactose; TX = Triton™ X-100; SA = Salicylic acid; NA = Not applicable; ND = Not determined; LSC = Lowest standard concentration; HSC = Highest standard concentration; LBC = Low bead count; '<' = Non-extrapolated values lower than LSC; '>' = Non-extrapolated values higher than HSC.

Notes:

1. Effect on viability from aerosol exposures to known irritants across a concentration range at 1-, 6-, and 24-hour timepoints in hPCLS tissues, compared to vehicle control set at 100%. Statistical analysis: *$p < 0.05$, student's t-test; 6 replicates per group.

2. The cytokine data from one or more tissues showing values outside the high and low standard concentration range were excluded from the analysis. The HSC and LSC values are averages of concentrations from standards run on 4 plates. * $p < 0.05$ compared to time-matched VC group; 2-way ANOVA with Dunnett's multiple comparison test; 3 replicates per group

3) A549 cell model

Figure S1. Dose-response curves for viability and IL-8 release at 24 hours in A549 cells.

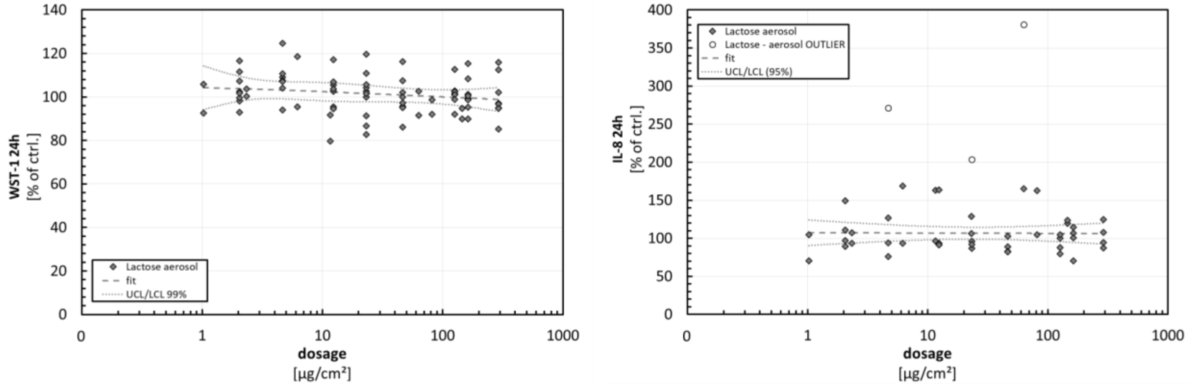

Figure S1a. Effect of **droplet aerosol exposure** with **lactose (negative control)** on A549 cultures at the ALI at 24h. No significant effects were observed up to 290 µg/cm². **Left:** Viability (WST-1) **Right:** IL-8 (ELISA).

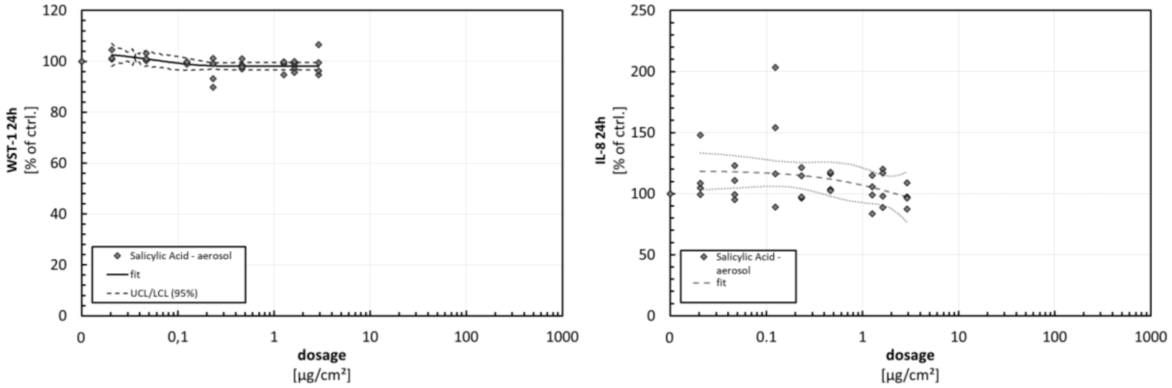

Figure S1b. Effect of **droplet aerosol exposure** with **salicylic acid (positive control)** on A549 cultures at the ALI at 24h. **Left:** Viability (WST-1); LOAEL: >2.91 µg/cm² **Right:** IL-8 (ELISA); LOA E L: <0.02 µg/cm².

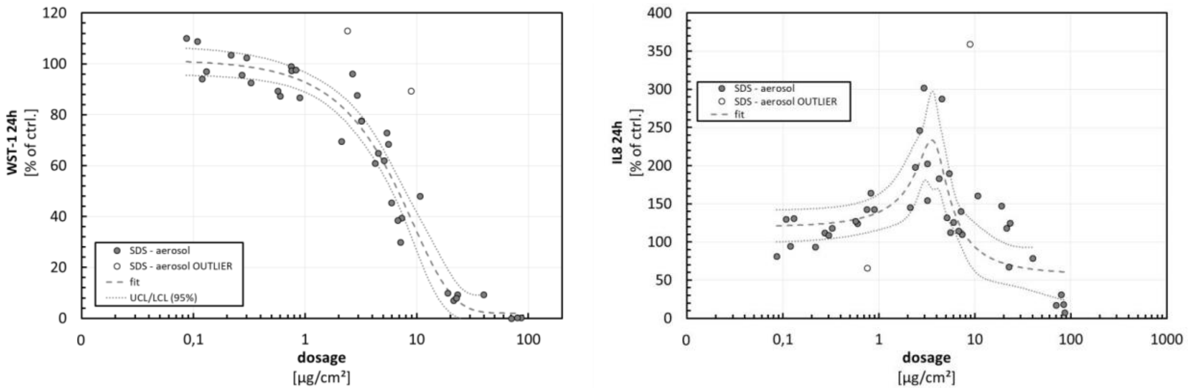

Figure S1c. Effect of **dry particle aerosol exposure** with **SDS (positive control)** on A549 cultures at the ALI at 24h. **Left:** The viability (WST-1); LOAEL: 0.65 µg/cm² **Right:** IL-8 (ELISA); LOAEL: 0.09 µg/cm²

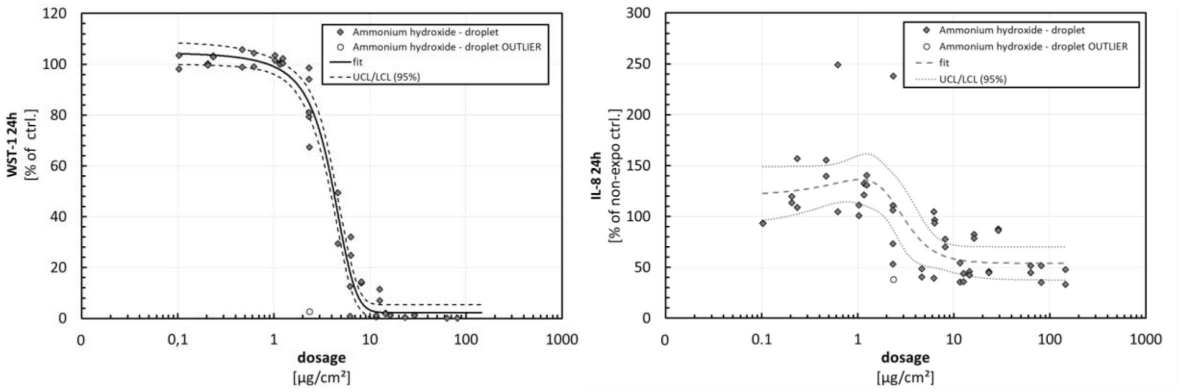

Figure S1d. Effect of **droplet aerosol exposure** with **Ammonium hydroxide** on A549 cultures at the ALI at 24h. **Left:** Viability (WST-1); LOAEL: 1.28 µg/cm² **Right:** IL-8 (ELISA); LOAEL: 0.18 µg/cm²

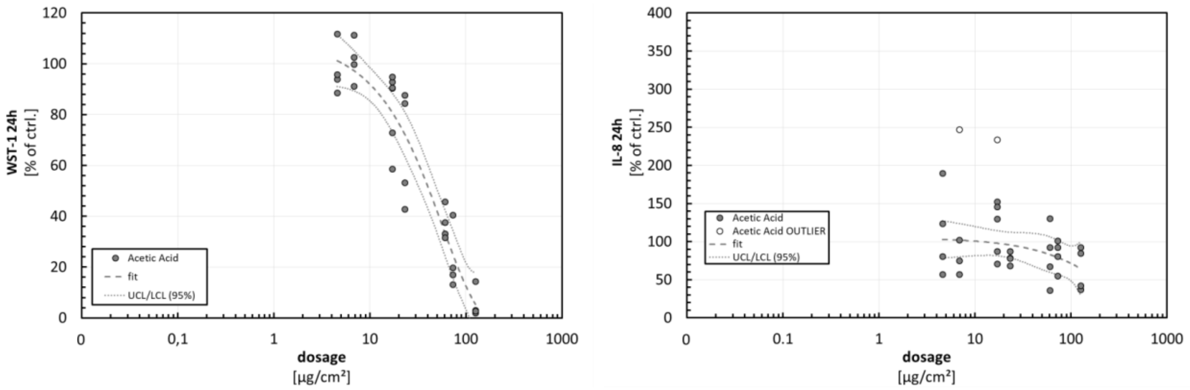

Figure S1e. Effect of **droplet aerosol exposure** with **Acetic acid** on A549 cultures at the ALI at 24h. **Left:** Viability (WST-1); LOAEL: 9.21 µg/cm² **Right:** IL-8 (ELISA); LOAEL: Not identified

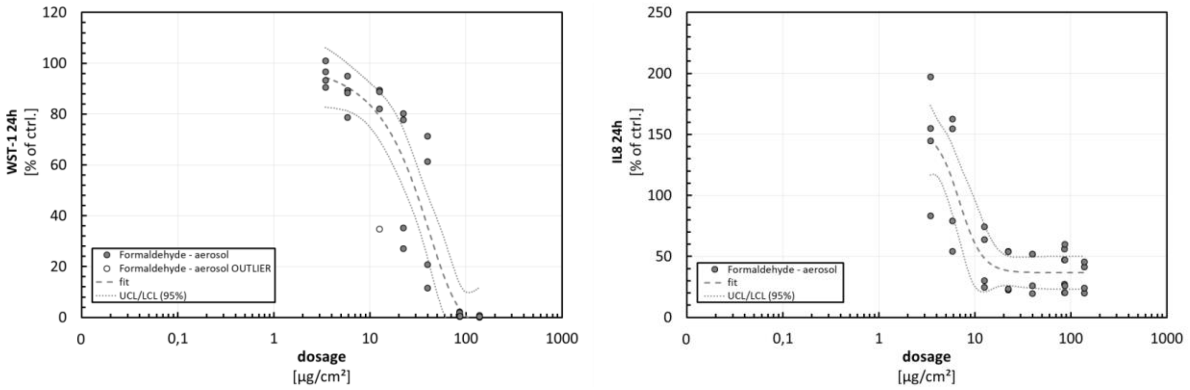

Figure S1f. Effect of **droplet aerosol exposure** with **Formaldehyde** on A549 cultures at the ALI at 24h. **Left:** Viability (WST-1); LOAEL: 5.82 µg/cm² **Right:** IL-8 (ELISA); LOAEL: <3.45 µg/cm² (lowest dose tested)

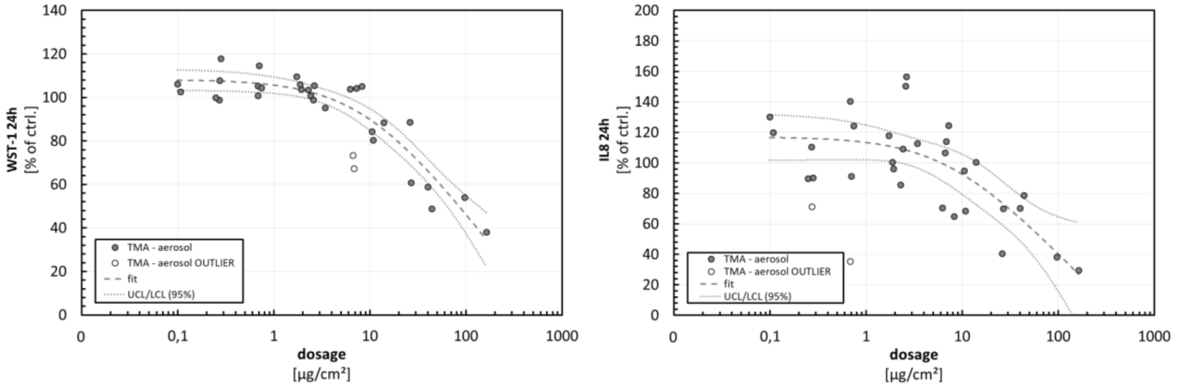

Figure S1g. Effect of **dry particle aerosol exposure** with **Trimellitic anhydride** on A549 cultures at the ALI at 24h. **Left:** Viability (WST-1); LOAEL: 5.88 µg/cm² **Right:** IL-8 (ELISA); LOAEL: <0.68 µg/cm² (lowest dose tested)

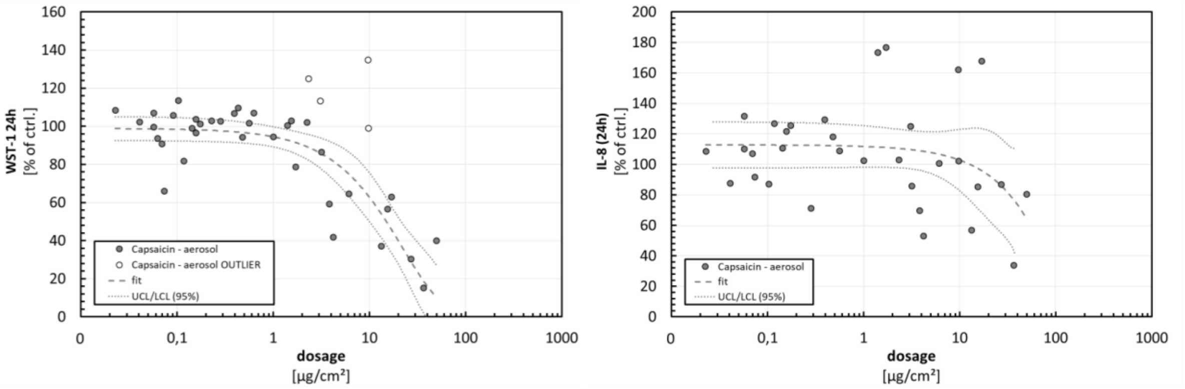

Figure S1h. Effect of **dry particle aerosol exposure** with **Capsaicin** on A549 cultures at the ALI at 24h. **Left:** Viability (WST-1); LOAEL: 0.94 µg/cm² (highest dose tested) **Right:** IL-8 (ELISA); LOAEL: Not detected

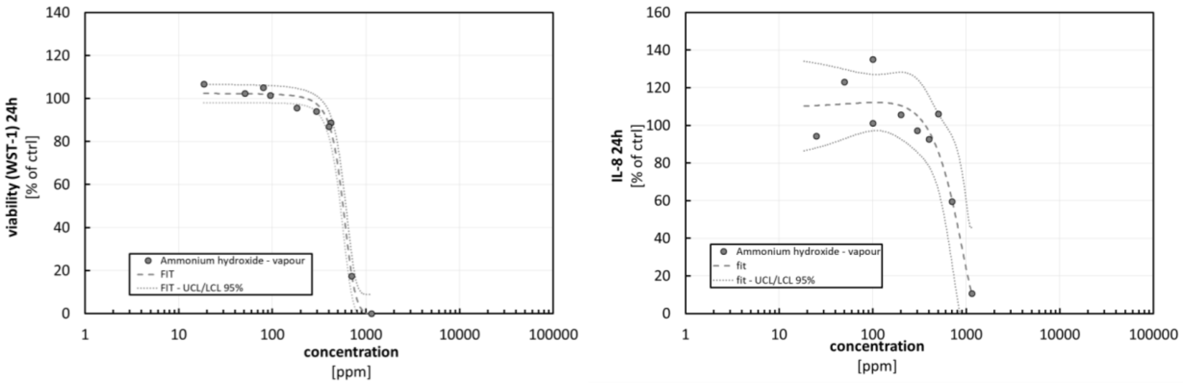

Figure S1i. Effect of **vapor exposure** with **Ammonium hydroxide** on A549 cultures at the ALI at 24h. **Left:** Viability (WST-1); LOAEL: 323 ppm **Right:** IL-8 (ELISA); LOAEL: Not detected

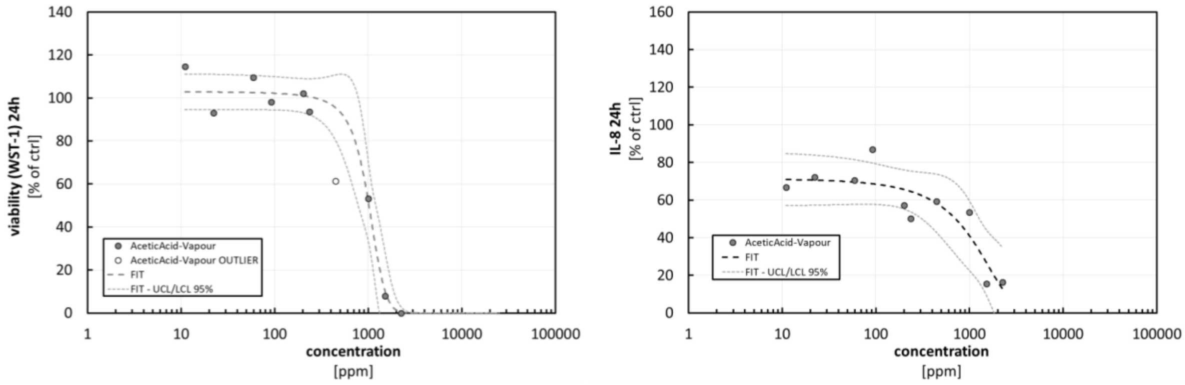

Figure S1j. Effect of **vapor exposure** with **Acetic acid** on A549 cultures at the ALI at 24h. **Left:** Viability (WST-1); LOAEL: 787.16 ppm **Right:** IL-8 (ELISA); LOAEL: Not detected

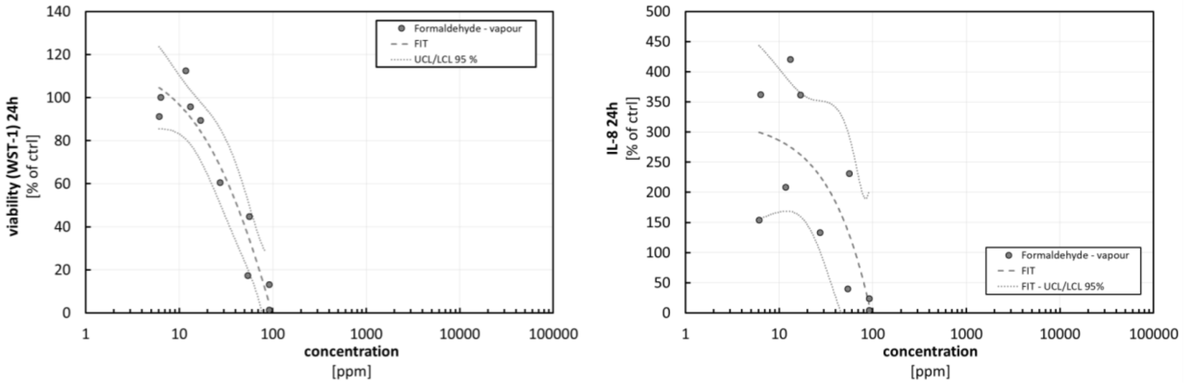

Figure S1k. Effect of **vapor exposure** with **Formaldehyde** on A549 cultures at the ALI at 24h. **Left:** Viability (WST-1); LOAEL: 15.34 ppm **Right:** IL-8 (ELISA); LOAEL: <6.1 ppm

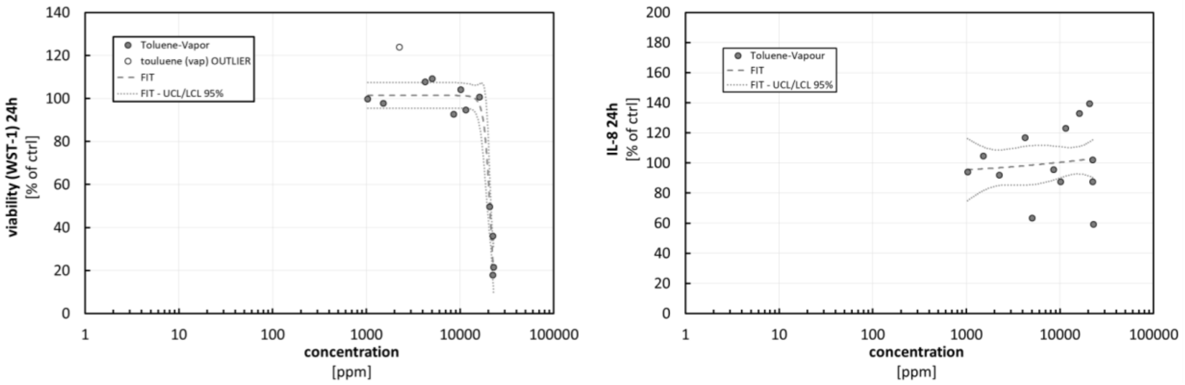

Figure S1l. Effect of **vapor exposure** with **Toluene** on A549 cultures at the ALI at 24h. **Left:** Viability (WST-1); LOAEL: 18862 ppm **Right:** IL-8 (ELISA); LOAEL: Not detected

Figure S2. Dose-response curves for mitochondrial membrane potential (MMP) and unspecified cellular stress measurements at 24 hours in A549 cells.

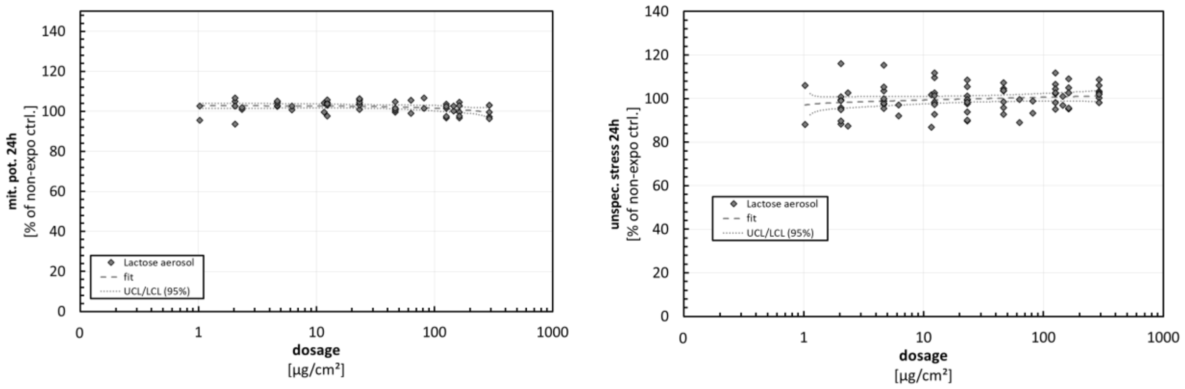

Figure S2a. Effect of **droplet aerosol exposure** with **lactose (negative control)** on A549 cultures at the ALI at 24h. No significant effects were observed up to 290 µg/cm². **Left:** MMP (JC-1); **Right:** Unspecific cellular stress (HOECHST)

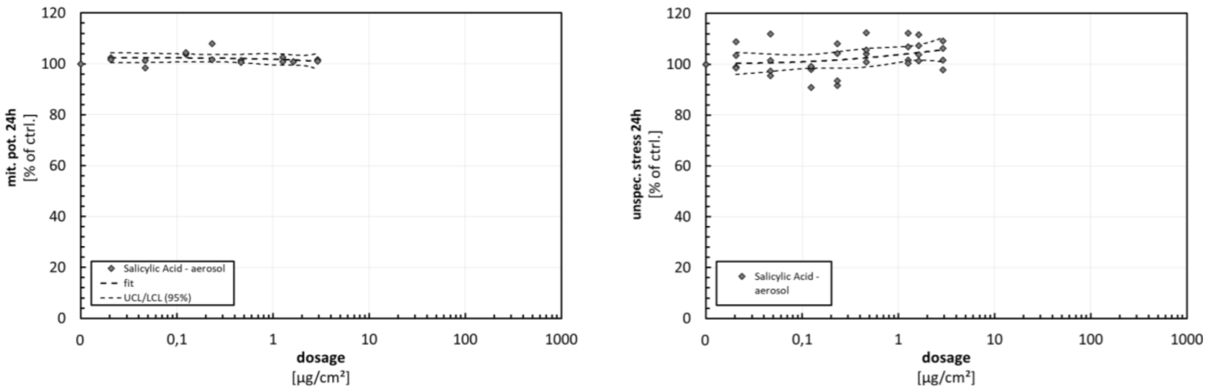

Figure S2b. Effect of **dry particle aerosol exposure** with **salicylic acid (positive control)** on A549 cultures at the ALI at 24h. **Left:** MMP (JC-1), LOAEL: >2.91 µg/cm²; **Right:** Unspecific cellular stress (HOECHST), LOAEL: 0.77 µg/cm²

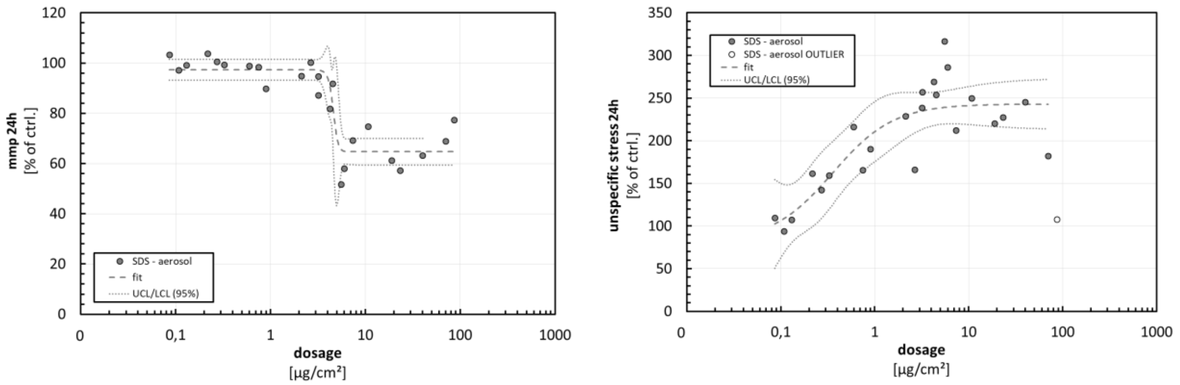

Figure S2c. Effect of **dry particle aerosol exposure** with **SDS (positive control)** on A549 cultures at the ALI at 24h. **Left:** MMP (JC-1), LOAEL: 4.97 µg/cm²; **Right:** Unspecific cellular stress (HOECHST), LOAEL: 0.22 µg/cm²

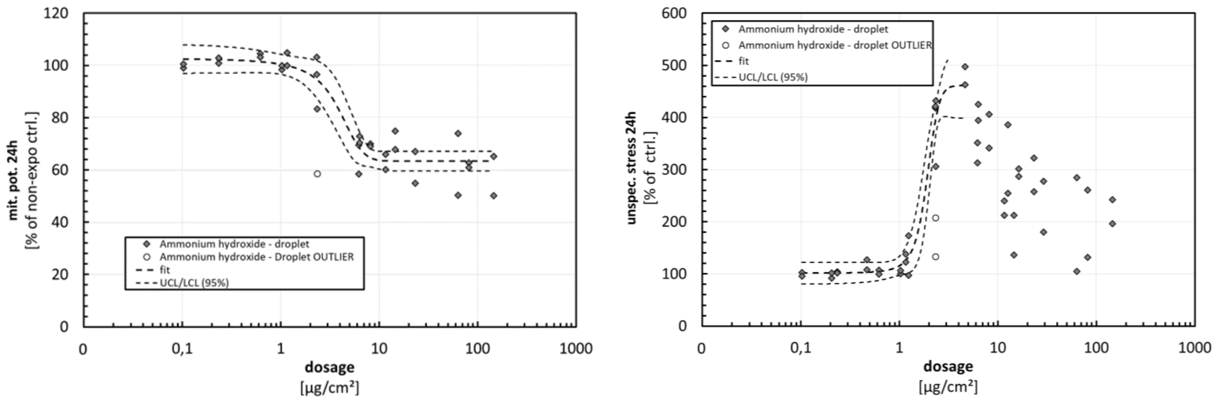

Figure S2d. Effect of **droplet aerosol exposure** with **Ammonium hydroxide** on A549 cultures at the ALI at 24h. **Left:** The mitochondrial membrane potential was measured by JC-1 live fluorescence staining. LOAEL: 2.83 µg/cm² **Right:** Unspecific cellular stress was measured by HOECHST live fluorescence staining. LOAEL: 1.16 µg/cm²

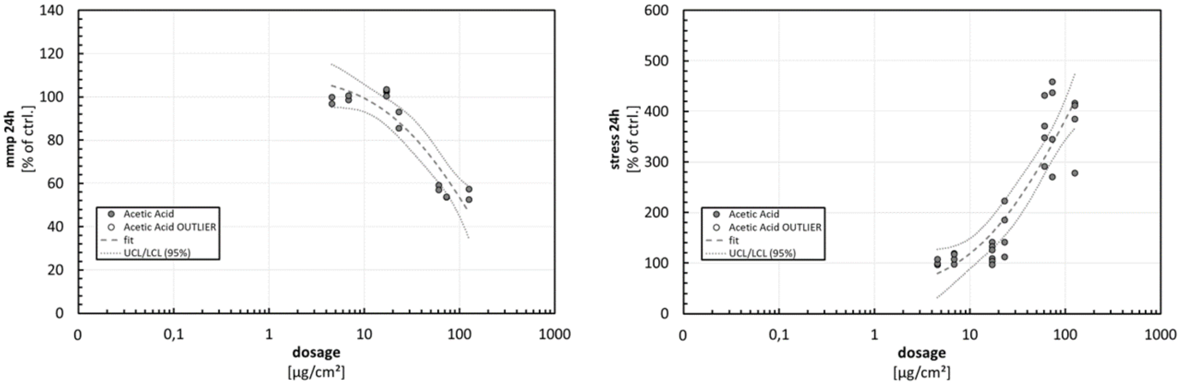

Figure S2e. Effect of **droplet aerosol exposure** with **Acetic acid** on A549 cultures at the ALI at 24h. **Left:** The mitochondrial membrane potential was measured by JC-1 live fluorescence staining. LOAEL: 16.04 µg/cm² **Right:** Unspecific cellular stress was measured by HOECHST live fluorescence staining. LOAEL: 11.7 µg/cm²

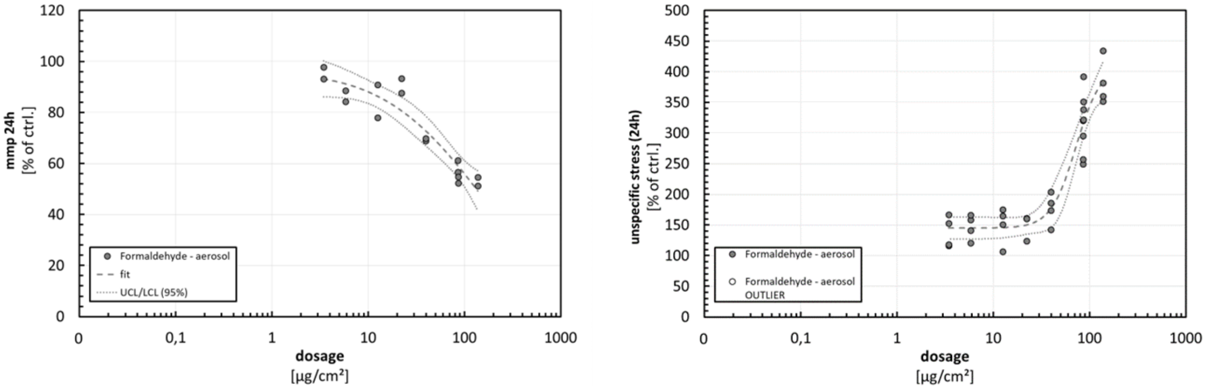

Figure S2f. Effect of **droplet aerosol exposure** with **Formaldehyde** on A549 cultures at the ALI at 24h. **Left:** The mitochondrial membrane potential was measured by JC-1 live fluorescence staining. LOAEL: <3.45 µg/cm² (lowest dose tested) **Right:** Unspecific

cellular stress was measured by HOECHST live fluorescence staining. LOAEL: <3.45 µg/cm² (lowest dose tested)

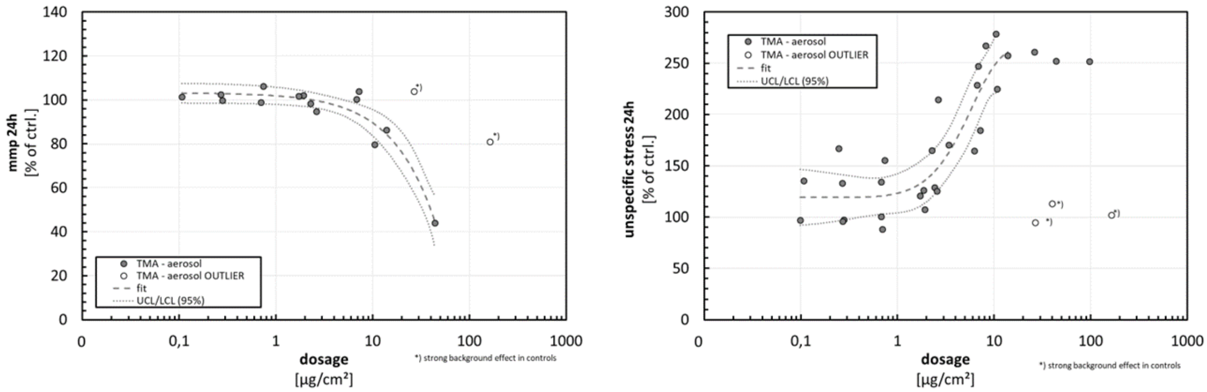

Figure S2g. Effect of **dry particle aerosol exposure** with **Trimellitic anhydride** on A549 cultures at the ALI at 24h. **Left:** The mitochondrial membrane potential was measured by JC-1 live fluorescence staining. LOAEL: 5.02 µg/cm² **Right:** Unspecific cellular stress was measured by HOECHST live fluorescence staining. LOAEL: 0.42 µg/cm²

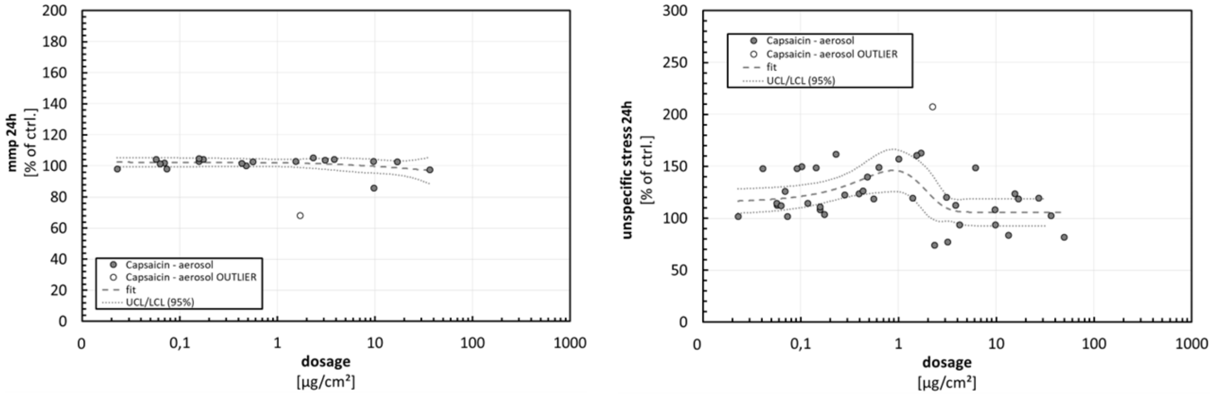

Figure S2h. Effect of **dry particle aerosol exposure** with **Capsaicin** on A549 cultures at the ALI at 24h. **Left:** The mitochondrial membrane potential was measured by JC-1 live fluorescence staining. LOAEL: >36.29 µg/cm² (highest dose tested) **Right:** Unspecific cellular stress was measured by HOECHST live fluorescence staining. LOAEL: <0.02 µg/cm² (lowest dose tested)

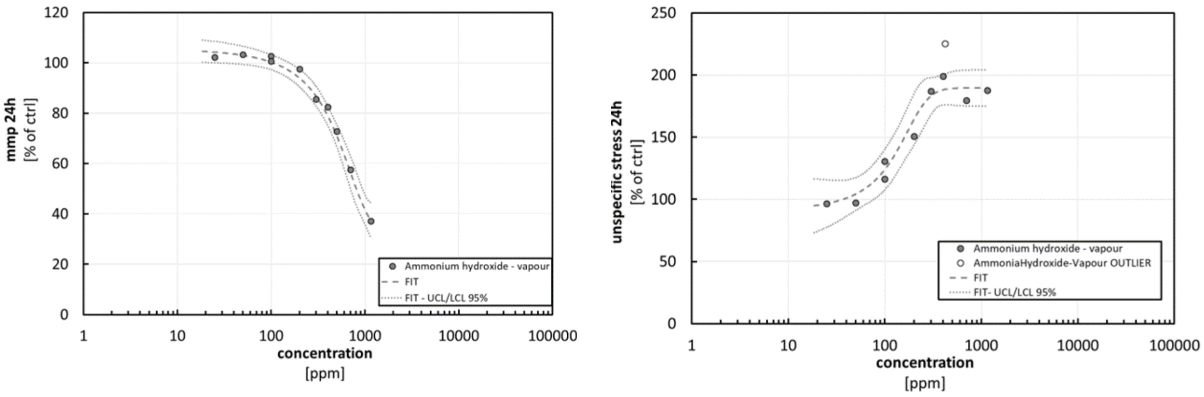

Figure S2i. Effect of **vapor exposure** with **Ammonium hydroxide** on A549 cultures at the ALI at 24h. **Left:** The mitochondrial membrane potential was measured by JC-1 live fluorescence staining. LOAEL: 157 ppm **Right:** Unspecific cellular stress was measured by HOECHST live fluorescence staining. LOAEL: 76.9 ppm

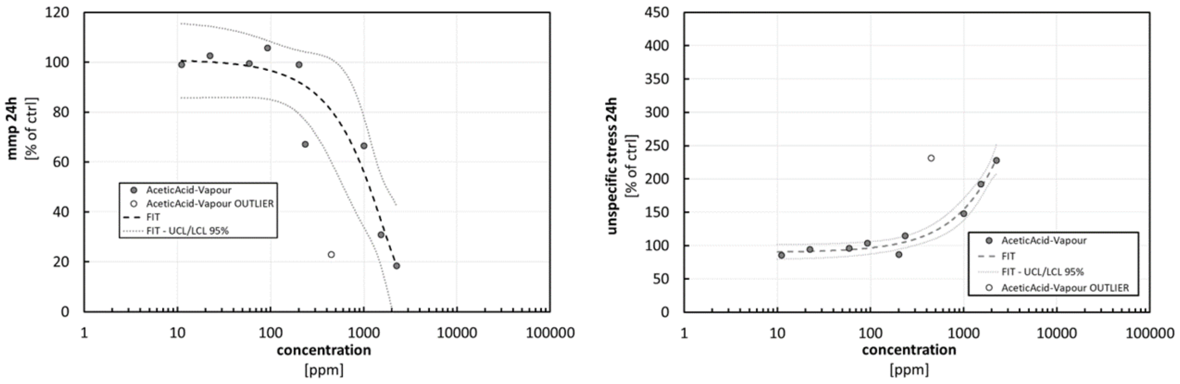

Figure S2j. Effect of **vapor exposure** with **Acetic acid** on A549 cultures at the ALI at 24h. **Left:** The mitochondrial membrane potential was measured by JC-1 live fluorescence staining. LOAEL: 501 ppm **Right:** Unspecific cellular stress was measured by HOECHST live fluorescence staining. LOAEL: 295 ppm

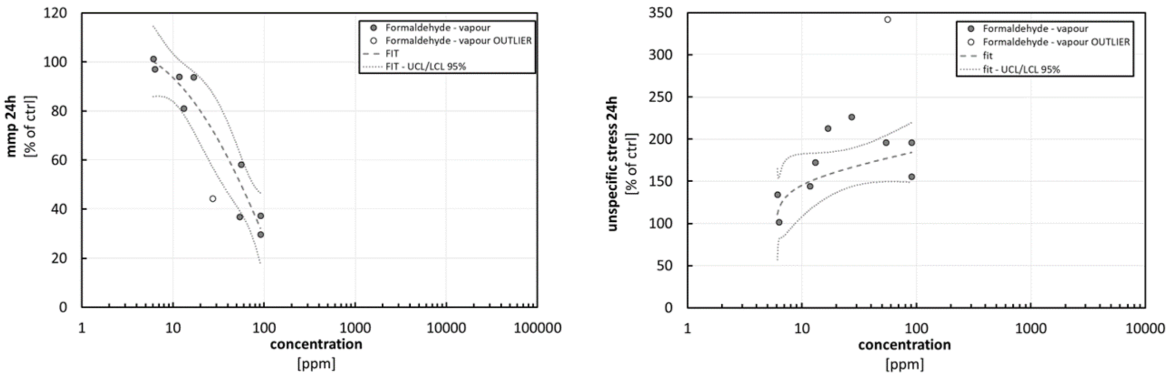

Figure S2k. Effect of **vapor exposure** with **Formaldehyde** on A549 cultures at the ALI at 24h. **Left:** The mitochondrial membrane potential was measured by JC-1 live fluorescence staining. LOAEL: 12.4 ppm **Right:** Unspecific cellular stress was measured by HOECHST live fluorescence staining. LOAEL: 8.7 ppm

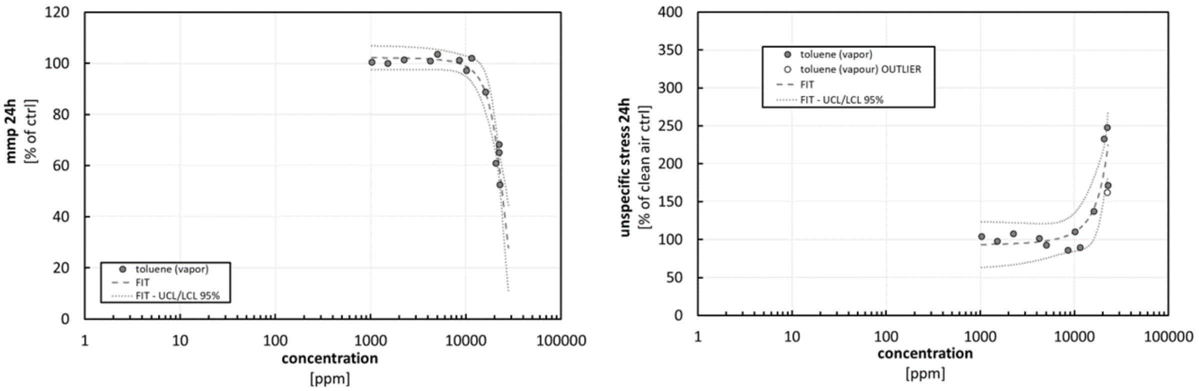

Figure S2l. Effect of **vapor exposure** with **Toluene** on A549 cultures at the ALI at 24h. **Left:** The mitochondrial membrane potential was measured by JC-1 live fluorescence staining. LOAEL: 26557 ppm **Right:** Unspecific cellular stress was measured by HOECHST live fluorescence staining. LOAEL: 16006 ppm
